# Mitfa-Independent Melanocyte Progenitors are Highly Susceptible to GNAQ-induced Uveal Melanoma in Adult Zebrafish

**DOI:** 10.1101/2025.05.05.652300

**Published:** 2025-09-09

**Authors:** Julius I. Yevdash, Delaney Robinson, Rachel Moore, Zhijie Li, Katelyn R. Campbell-Hanson, Danielle Gutelius, Stephen P. G. Moore, Dylan Friend, Isaac O’Toole, Collin Montgomery, Jesse D. Riordan, Adam J. Dupuy, Robert A. Cornell, Elaine M. Binkley, Deborah Lang, Ronald J. Weigel, Colin Kenny

**Affiliations:** 1. Department of Surgery, College of Medicine, University of Iowa, Iowa City, IA 52242, USA.; 2. Department of Dermatology, Boston University School of Medicine, Boston, MA, 02118, USA.; 3. Department of Anatomy & Cell Biology, University of Iowa, Iowa City, IA, 52242, USA.; 4. Holden Comprehensive Cancer Center, University of Iowa, Iowa City, IA 52242, USA.; 5. Department of Oral Health Sciences, University of Washington, School of Dentistry, Seattle, WA, 98195; 6. Department of Ophthalmology and Visual Sciences, University of Iowa, Iowa City, IA 52242, Iowa; 7. University of Iowa, Institute for Vision Research, Iowa City, IA 52242, Iowa; 8. Lead Contact.

## Abstract

Melanocytes reside in diverse microenvironments that influence their susceptibility to oncogenic transformation, however, studying rare melanoma subsets has been hindered by the lack of suitable animal models. We developed a primary, immune-competent zebrafish model to study uveal melanoma (UM), utilizing choroidal-targeted injection and electroporation of plasmids containing human *GNAQ*^*Q209L*^ and CRISPR/Cas9 cassettes for tumor suppressor gene deletion. Single-cell transcriptional profiling of genetically identical eye- and skin-derived tumors revealed distinct oncogenic pathways, highlighting the importance of studying melanoma subtypes in their correct anatomical context. Additionally, we identified a population of *tfec*- and *pax3a*-expressing melanocyte progenitor cells in *mitfa*-deficient embryos and adult zebrafish eyes, which were highly susceptible to GNAQ-driven transformation. While previous studies have linked *mitfa* deficiency to accelerated UM onset, our findings suggest that an expanded progenitor population in *mitfa*-deficient animals drives this susceptibility. Our study establishes a critical role for Mitfa-independent melanocyte progenitors in UM pathogenesis

## Introduction

Uveal melanoma (UM) is a rare cancer of the melanocytes in the eye that is both vision threatening and deadly.^1-4^ The majority of UM’s (~90%) occur in the choroid, the vascular structure supplying the outer retina, with the remainder arising from the ciliary body or the iris.^5-9^ Oncogenic mutations in *GNAQ* or its paralog *GNA11* are observed in up to 90% of UMs, but are rare in cutaneous melanoma (CM).^4,10^ Such mutations reduce GTPase activity of the heterotrimeric G protein alpha subunit, resulting in constitutive activation that drives cellular proliferation through downstream signaling pathways, including MAPK, YAP/TAZ, and PI3K signaling.^5,11-13^

UM and CM display distinct metastatic progression. Up to 50% of UM tumors metastasize to the liver via hematogenous spread,^3,14,15^ and survival after metastasis is generally poor ^16^ There remain few FDA approved options for patients with metastatic UM. Tebentafusp was the first systemic therapy shown to improve overall survival in metastatic UM at both 1 year and 3 years; however, this is only approved in HLA-A02:01-positive patients and, despite being an improvement, progression free survival at 6 months was only 31%, and median overall survival at 3 years was only 21.6 months in treated patients ^17,18^ Chemotherapy and immune checkpoint inhibitors, which are highly effective in CM, have very limited efficacy in UM.^19,20^ Some promising targeted therapies are currently under investigation, including Darovasertib, a PKC inhibitor targeting GNAQ/GNA11-driven MAPK signaling,^21^ and more invasive options, such as melphalan-based liver treatment, have demonstrated some benefit in the absence of other effective systemic options.^22^

While *GNAQ/11* oncogenic mutations are uncommon in CM, the majority of CMs harbor activating mutations in *BRAF* or *NRAS*.^5,23-25^ This distinction suggests that unique properties of melanocytes in different tissues, or perhaps their tumor microenvironments (TME), may drive differential oncogene sensitivity. Melanocytes in the choroid interact with fibroblasts and vasculature endothelial cells, whereas melanocytes in the skin are in direct contact with keratinocytes. In transgenic experiments, BRAF^V600E^-positive melanocytes exhibit significantly greater survival in the epidermis compared to GNAQ^Q209L^-positive melanocytes. However, GNAQ^Q209L^-positive melanocytes preferentially survived in the dermis, where fibroblasts are abundant.^26^ This suggests that oncogene-specific interactions with the microenvironment influence melanocyte survival and provide a basis for the absence of *GNAQ* mutations in CM, except for dermal blue nevi which interact closely with fibroblasts.^10,26^ Interestingly, while UM and CM are unique in their metastatic patterns, GNAQ/11-mutant non-uveal melanoma demonstrates a lymphatic metastatic progression that resembles CM, thus further indicating the importance of studying UM in the correct anatomical context.^24^

Zebrafish are a well-established model for studying melanocytes and melanoma due to conserved melanocyte differentiation pathways, efficient genetic manipulation, and the use of transparent lines that facilitate high-resolution live-cell imaging.^24,27-35^ Zebrafish have three types of pigment cells; dark melanocytes, iridescent iridophores, and yellow xanthophores, that arise from multipotent, neural crest-derived pigment progenitors.^24,33,36-39^ Melanomas have been shown to upregulate neural crest transcriptional programs, which are associated with worse clinical outcomes.^29,40-42^ Similarly, *Tg(mitfa:GNAQ*^*Q209L*^*); mitfa*^*−/−*^ zebrafish, which retain melanocyte progenitor cells but lack differentiated melanocytes,^43^ have earlier occurrences of UM than wild-type zebrafish.^34^ By contrast, BRAF-driven CM displays a reliance on Mitfa activity to progress.^44,45^ To study the early events of UM, we developed a novel zebrafish model that utilizes the transgene electroporation of adult zebrafish (TEAZ) approach to express the human GNAQ^Q209L^ oncogene in cells of the melanocyte lineage within the skin and eye. By targeting injections to the choroid, primary UM tumors arise from the same anatomical location as human UM, recapitulating similar TME and initiating cell types. In this study, we analyze expression differences between genetically identical skin and eye-derived GNAQ^Q209L^ tumors and demonstrate the existence of a melanocyte precursor population in the eyes zebrafish that have an increased sensitivity to transformation by GNAQ^Q209L^.

## Results

### Generation of plasmid constructs to drive GNAQ^Q209L^-induced melanoma.

**Figure 1:**
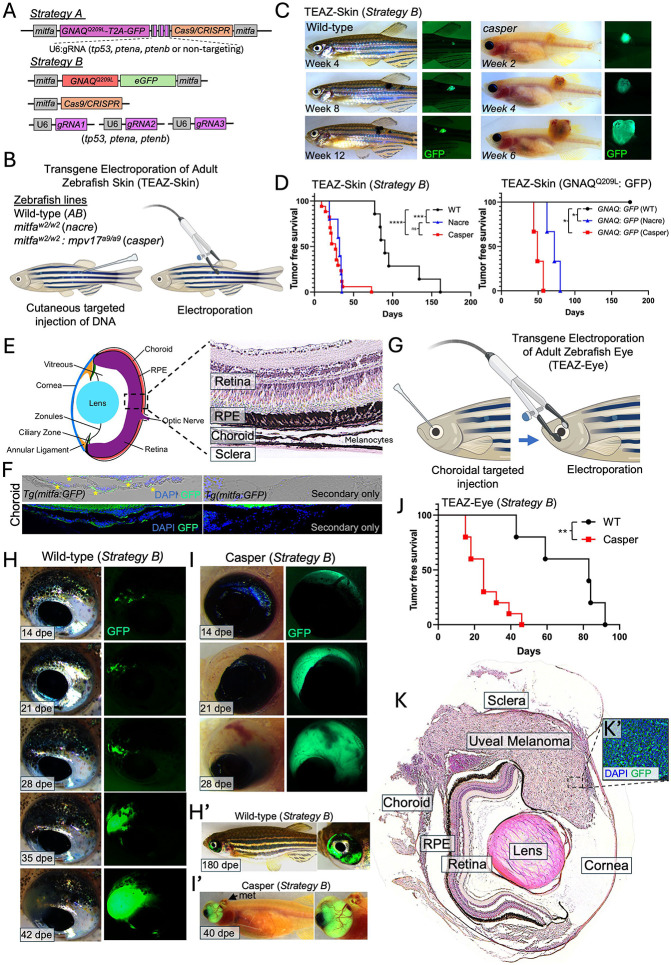
*GNAQ*^Q209L^-induced melanoma by TEAZ-Skin and TEAZ-Eye. (**A**) TEAZ-Skin was used to induce GNAQ-positive melanoma using two strategies. Strategy A involves a single plasmid construct containing expression cassettes driven by the *mitfa* promoter for GNAQ^Q209L^-T2A-eGFP and Cas9, as well as a U6 promoter driving the expression of three separate gRNAs (targeting *tp53, ptena*, and *ptenb*, or a non-targeting control). Strategy B uses a multi-plasmid approach with five plasmids for TEAZ-Skin. Expression cassettes include: (a) *mitfa*:*GNAQ*^Q209L^-*mitfa*:eGFP, (b) *mitfa*:Cas9, (c) *U6*:gRNA (*tp53*), (d) *U6*:gRNA (*ptena*), and (e) *U6*:gRNA (*ptenb*). (**B**) Schematic of TEAZ-Skin using wild type (*AB*), *nacre* (*mitfa^w2/w2^*) and *casper* (*mitfa^w2/w2^: mpv17^a9/a9^*) zebrafish lines. (**C**) TEAZ-Skin using Strategy B plasmids in adult wild-type (*AB*) and *casper* zebrafish. Representative brightfield and fluorescent images shown at 4, 8, and 12 weeks post electroporation for WT and at 2, 4, and 8 weeks for *casper*. (**D**) Kaplan-Meier curves were used to estimate tumor-free survival, defined as the time from electroporation until the first visible appearance of GFP+ nodular tumors, in adult zebrafish via TEAZ-Skin. Strategy B wild-type (n=7), *nacre* (n=5), and *casper* (n=17) curves and TEAZ-Skin (*GNAQ*^Q209L^:GFP) curves (n=3 per condition) compared pairwise with logrank (Mantel-Cox) test. ns = not significant, * p-value<0.05; ** p-value<0.01; ***p-value<0.001; ****p-value<0.0001. (**E**) Schematic of the zebrafish eye, dashed box indicates the retina and choroid. Hematoxylin and eosin (H&E) staining of a wild-type zebrafish eye at 20× magnification, showing the retina and choroid. Eye structures are labeled, including choroidal melanocytes. (**F**) Immunofluorescent analysis of transgenic *Tg(mitfa:GFP)* zebrafish using an anti-GFP antibody. Sections were counterstained with DAPI to visualize nuclei. Representative image showing GFP expression in *mitfa*-positive cells. Secondary-only antibody was used as background control. (**G**) Schematic of TEAZ-Eye using choroidal targeted injection. (**H-H’**) Live imaging of adult wild-type zebrafish following TEAZ-Eye with Strategy B plasmids to induce uveal melanoma. Brightfield imaging and the corresponding GFP fluorescence images highlighting tumor progression. Timepoints in day post-electroporation (dpe) as labeled. (**I-I’**) Live imaging of adult *casper* zebrafish over 21 days post-electroporation (dpe) using TEAZ-Eye with Strategy B plasmids to induce uveal melanoma. Brightfield imaging and the corresponding GFP fluorescence images highlighting tumor progression. (**J**) Kaplan-Meier curves comparing tumor-free survival among wild-type (n=5) and *casper* (n=10) zebrafish following TEAZ-Eye induction using Strategy B plasmids. Curves compared pairwise with logrank (Mantel-Cox) test. ** p-value<0.01. (**K, K’**) H&E analysis of uveal melanoma in *casper* zebrafish induced by TEAZ-Eye. Images were acquired at 20× magnification and stitched from 85 fields using Thunder imaging software. Normal eye tissue and uveal melanoma as labeled. (**K’**) Representative 40x magnification image showing GFP expression in tumors cells after anti-GFP immunofluorescent analysis of TEAZ-Eye induced uveal melanoma in *casper* zebrafish.

To study GNAQ-driven melanoma in adult zebrafish, we generated expression plasmids containing two cassettes under the control of the *mitfa* promoter. The first cassette encoded human oncogenic *GNAQ*^*Q209L*^ fused to a T2A self-cleaving peptide followed by eGFP, while the second cassette expressed CRISPR/Cas9. Additionally, three zebrafish U6 promoters were used to drive gRNAs targeting *tp53* and *ptena/b* (Figure 1A – strategy A). Recognizing that T2A cleavage results in residual amino acids remaining on the C-terminus of GNAQ, which may alter its oncogenic activity, we employed an alternative delivery strategy. This system utilized five separate plasmids: (1) *mitfa:GNAQ*^*Q209L*^*; mitfa:eGFP* (a generous gift from Dr. Jacqueline Lees at the KOCH institute Massachusetts Institute of Technology), (2) *mitfa:Cas9*, (3) *U6:*gRNA-*tp53*, (4) *U6:*gRNA-*ptena* and (5) *U6:*gRNA-*ptenb* (Figure 1A – strategy B) (plasmids 2-5 were a generous gift from Dr. Richard White at the University of Oxford, UK). This design aimed to improve the oncogenicity of GNAQ^*Q209L*^ by avoiding unintended C-terminal modifications by T2A cleavage. Moreover, the plasmids used in strategy B are smaller in size, which may enhance the efficiency of uptake during electroporation.

We applied TEAZ to the skin (TEAZ-Skin) of adult wild-type (*AB*), *mitfa*^*w2/w2*^ (*nacre* ^43^), and *mitfa*^*w2/w2*^*; mpv17*^*a9/a9*^ (*casper*^46,47^) zebrafish to validate the oncogenic potential of *GNAQ*^*Q209L*^ (Figure 1B). TEAZ-Skin using Strategy A and Strategy B induced melanoma in wild-type zebrafish within an average of 147- and 104-days, respectively (Figure 1C-D, Figure S1A). TEAZ-Skin using plasmids to express oncogenic *GNAQ* alone or Cas9 with gRNAs to delete *tp53, ptena* or *ptenb* alone did not induce melanoma, suggesting that both oncogenic *GNAQ* and tumor suppressor deletion are required for transformation (Figure S1A, black line, n=15). TEAZ-Skin was subsequently applied to zebrafish lacking functional *mitfa* (*nacre* and *casper*), which lack differentiated melanocytes. Consistent with previous observations in transgenic *Tg(mitfa-GNAQ*^*Q209L*^*); mitfa*^*w2/w2*^*; tp53*^*−/−*^ zebrafish,^34^ *mitfa* loss resulted in decreased tumor latency compared to wild-type zebrafish (Figure 1C-D, Figure S1B,B’-C). Tumors were detected within an average of 30- and 28-days post-electroporation following Strategy B in *nacre* and *casper*, respectively. Moreover, unlike wild-type zebrafish and consistent with previous reports,^40^ expression of oncogenic GNAQ alone was sufficient to induce melanoma in *nacre* (Figure 1D, Figure S1B) and *casper* zebrafish (Figure 1D, Figure S1C-D). We ruled out xanthomas as the cell of origin for TEAZ-Skin tumors, given the distinct visual differences between the tumors and the xanthomas observed in *Tg*(*mitfa*^w2/w2^, *mitfa*:*BRAF*^*V600E*^, *tp53*^−/−^) zebrafish. The xanthomas were bright orange, opaque, and clearly distinguishable from the darkly pigmented UM tumors in wild-type zebrafish and the beige, translucent UM tumors in *casper* zebrafish (Figure S1E). CRISPR editing in TEAZ-Skin tumors was confirmed by Sanger sequencing. All but one tumor (10 of 11) exhibited edits in all three targeted tumor suppressors (*tp53*, *ptena*, and *ptenb*), with the remaining tumor lacking *ptena* mutation. As expected, uninjected tissue showed no evidence of CRISPR editing (Figure S2).

Together, our findings demonstrate that both TEAZ-Skin strategies are sufficient to induce GNAQ-driven melanoma, with strategy B demonstrating reduced tumor latency compared to strategy A. Notably, TEAZ-Skin accelerates melanoma onset relative to transgenic models;^34^ while tumors in transgenic *Tg(mitfa:GNAQ*^*Q209L*^*); tp53*^*−/−*^ zebrafish typically arise 200–400 days post-fertilization,^34^ TEAZ-Skin induces UM tumors by approximately 100 days in wild-type zebrafish.

### The *mitfa* promoter is active in choroidal melanocytes

Having confirmed the oncogenicity of our *GNAQ*^*Q209L*^ expression plasmids using TEAZ-Skin, we next sought to adapt this approach to induce melanocyte transformation in the zebrafish eye (TEAZ-Eye), specifically targeting the choroid, where the majority of ocular melanocytes reside (Figure 1E). To determine whether TEAZ-Eye could serve as a model for UM in adult zebrafish, we first assessed the specificity of the *mitfa* promoter in driving transgene expression in choroidal melanocytes. To this end, we harvested eyes from transgenic *Tg(mitfa:GFP)* zebrafish and performed immunofluorescent imaging using an anti-GFP antibody. Choroidal melanocytes exhibited strong anti-GFP positivity compared to the secondary-only control (Figure 1F). Of note, the retinal pigment epithelium (RPE), located apically to the choroidal melanocytes, contains autofluorescent granules. Quenching the autofluorescence signal from the RPE improved the anti-GFP signal-to-noise ratio (Figure S3A-B). We next applied TEAZ-Eye by injecting and electroporating plasmids containing the *mitfa* promoter driving *eGFP* expression (*mitfa:eGFP*) into the choroid. Eyes were harvested two weeks post-electroporation, and immunofluorescence using an anti-GFP antibody was performed. Remarkably, cells at the injection site exhibited a strong eGFP signal compared to the secondary-only control (Figure S3C). These results demonstrate that the *mitfa* promoter is sufficient to drive transgene expression in choroidal melanocytes using TEAZ-Eye.

### TEAZ-Eye induces UM in the choroid of wild-type and *casper* zebrafish

A major limitation in the study of UM onset and progression is the lack of anatomically accurate *in vivo* models. For instance, UM tumors derived in transgenic *Tg(GNAQ*^*Q209L*^*): tp53*^*−/−*^ zebrafish predominantly form in the skin, with ~1–2% of tumors forming in the eye.^34^ To address this gap, we applied TEAZ-Eye using a plasmid pool containing *mitfa:GNAQ*^*Q209L*^*, mitfa:Cas9, U6:*gRNA*-tp53, U6:*gRNA*-ptena, and U6:*gRNA*-ptenb* (i.e., Strategy B) into the choroidal space of wild-type zebrafish, followed by electroporation (Figure 1G-H). Remarkably, TEAZ-Eye in wild-type zebrafish resulted in the transformation of cells within the choroid. Live *in vivo* imaging of TEAZ-Eye enabled the identification of single GFP+ cells at the injection site, serving as a proxy for *GNAQ*^*Q209L*^ expression (Figure 1H-H’). We next applied TEAZ-Eye to *mitfa*-deficient *casper* zebrafish (Figure 1I-I’), and like with TEAZ-Skin, tumor latency of TEAZ-Eye injected *casper* zebrafish (n=10) was significantly reduced compared to wild-type (n=5) (Figure 1J). H&E analysis confirmed that TEAZ-Eye induced UM within the choroid, progressing into surrounding ocular structures (Figure 1K). Notably, 100% (n=10) of tumors formed between the sclera and RPE, highlighting the model’s ability to induce anatomically accurate UM. Finally, immunofluorescent analysis using anti-GFP antibodies confirmed the expression of plasmid constructs following TEAZ-Eye (Figure 1K’).

### UM transformation involves activation of neural crest transcriptional programs

**Figure 2:**
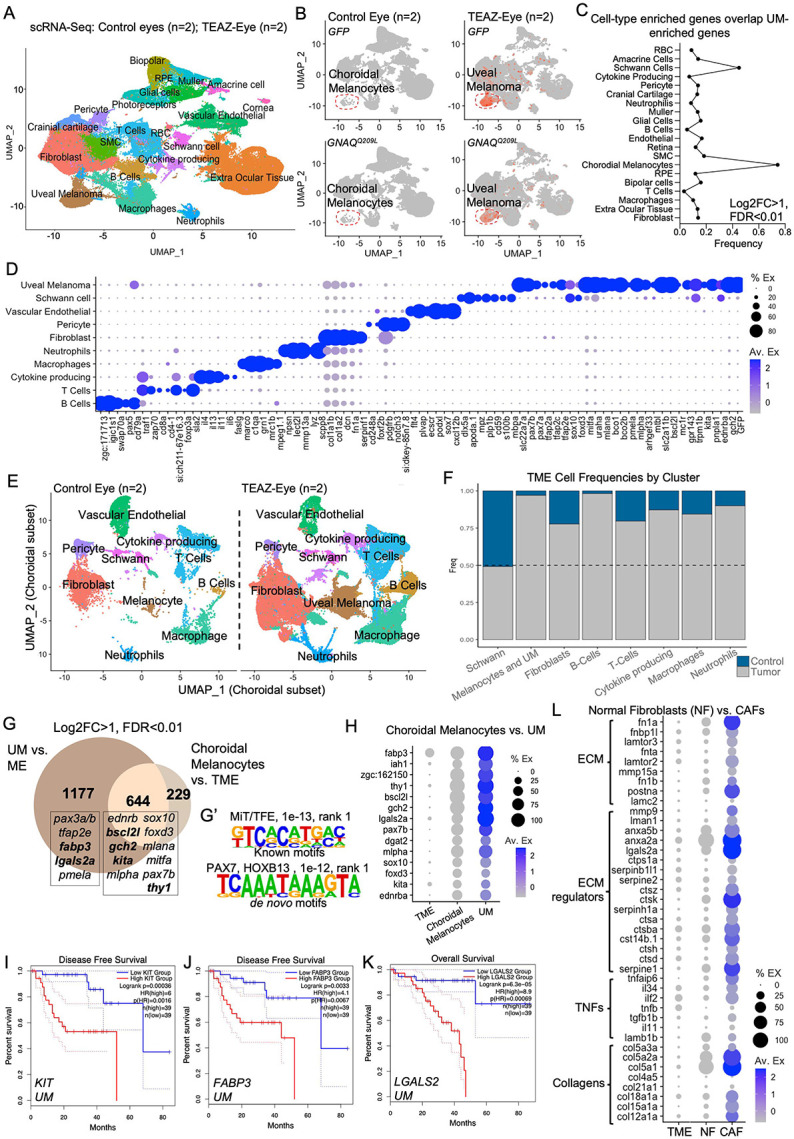
Single cell profiling of uveal melanoma and the TME in adult zebrafish. (**A**) UMAP representation of single-cell RNA-Seq (scRNA-seq) data from three dissociated wild-type eyes harboring uveal melanoma (UM) that were generated by TEAZ-Eye (sequencing replicates, n=2), and control eyes consisting of four pooled sibling-matched wild-type eyes, and two tumor paired control eyes (sequencing replicates, n=2). Annotated cell clusters as labeled. (**B**) UMAP and feature plot showing *eGFP* and *GNAQ*^Q209L^ expression in control eyes and UM tumor. Red dotted line highlights the melanocyte and UM clusters. (**C**) Comparison of UM tumor marker genes with the markers of cell types within the control eye microenvironment. Only genes with a log2FC > 1 and q-value < 0.01 were included in the analysis. (**D**) Dot plot representing enriched genes (log2FC > 1; q-value < 0.01) in choroidal cell populations, UM tumors cells, immune cell populations, fibroblasts, Schwann cells, pericytes, and vascular endothelial cells as shown. Dot size indicates the percentage of cells expressing each gene; color intensity (grey to blue) reflects normalized average expression levels (low to high). (**E**) UMAP clustering of cells in the choroidal microenvironment. Annotated cell clusters as labelled. (**F**) Comparison of relative cluster sizes between the two conditions, control and TEAZ-Eye, shown in (E). Schwann cells serve as a control cluster, displaying no change in frequency between conditions. Gray = TEAZ-Eye, Blue = Control. (**G**) Venn diagram representing the overlap of genes between UM tumors and choroidal melanocytes compared to the cells in their respective microenvironments. Enriched genes for each condition were identified by differential expression analysis comparing melanocytes to the microenvironment (ME) or tumor cells to the tumor microenvironment (TME), using a threshold of log2FC > 1 and q-value < 0.01. Genes of interest have been labelled in their respective subsets. (**G’**) HOMER promoter motif enrichment analysis of genes enriched in UM tumors vs TME. The top ranked motif for “known” and *“de novo”* motifs are shown with their corresponding p-values. (**H**) Dot plot showing enriched genes (log2FC > 1; q-value < 0.01) in UM tumor cells, choroidal melanocytes, or the tumor microenvironment (TME). Dot size indicates the percentage of cells expressing each gene; color intensity (grey to blue) reflects normalized average expression levels (low to high). (**I-K**) Kaplan–Meier disease-free survival and overall survival curves generated using uveal melanoma TCGA data. Patients stratified by relative expression levels of *KIT, FABP3*, or *LGALS2*. Gene expression thresholds were defined by median expression within each cohort. Log rank p-values as shown. Dotted lines represent 95% confidence interval. (**L**) Dot plot showing enriched genes (log2FC > 1; q-value < 0.01) in cancer-associated fibroblasts (CAF), normal fibroblasts (NF), or the tumor microenvironment (TME) without CAF. Dot size indicates the percentage of cells expressing each gene; color intensity (grey to blue) reflects normalized average expression levels (low to high). Genes have been grouped according to their labelled functions.

During embryonic development, choroidal melanocytes are derived from the cranial neural crest (CNC)^48^. Since our model can detect the earliest events in UM transformation, we asked whether the reactivation of CNC programs is one of the initial steps in UM development. To achieve this goal, tumors were derived in wild-type zebrafish using TEAZ-Eye with Strategy B plasmids. At 50–90 dpe, when gross nodular tumors were identified, surgical enucleation was performed, and dissociated cells from the entire eye were subjected to single-cell RNA sequencing (scRNA-seq). We compared pooled wild-type tumors (2 sequencing replicates) with tumor paired bilateral eyes or sibling-matched control eyes (2 and 4 pooled eyes, respectively, 2 sequencing replicates). Given that whole eyes were used as control samples and melanocytes are most abundant in the choroid, melanocytes identified under control conditions are hereafter referred to as choroidal melanocytes. Tumor cells were identified by expression of *GFP* and oncogenic *GNAQ* (Figure 2B) whereas primary choroidal melanocytes were marked by expression of *mlana* (Figure S4). Annotation of additional cell types within the TME was accomplished by comparing to previously published scRNA-seq datasets using human choroid and retina,^49,50^ the Zebrafish Information Network (ZFIN),^51^ and from the zebrafish embryo single cell atlas “Daniocell”^52^ (Figure 2A). Interestingly, tumor cells aligned most closely with choroidal melanocytes, ruling out the retinal pigmented epithelium (RPE) as an origin of our TEAZ-Eye induced tumors (Figure 2C). Additionally, profiling *GFP/GNAQ*^*Q209L*^+ UM cells revealed that tumors exhibited strong expression of neural crest markers (*tfap2a, tfap2c, pax7b, sox10, foxd3*) and melanocytic markers (*mitfa, tfap2e, mlana, pmela, slc22a7a, mlpha, mtbl*) (Figure 2D). Unexpectedly, the CNC marker genes *twist1* or *dlx1*^53,54^ were not enriched in UM tumors compared to the TME. Pathway analysis showed that the genes enriched in TEAZ-Eye-derived tumors were strongly associated with MITF-M-dependent gene expression, RAF/MAP kinase, mTOR, PI3K/AKT signaling, NF-kB, RHO GTPase, and NOTCH activity, as well as fatty acid oxidation pathways (Figure S6A-B).

Re-clustering of choroidal cells from control and tumor conditions revealed a marked expansion of tumor cells relative to melanocytes which was accompanied by increases in immune cell populations and fibroblasts (Figure 2E-F). Immune cell populations within the TME included B-cells (*igic1s1, pax5, cd79a, zgc:153659*), T-cells (*traf1, si:ch211–67e16.3, cd27, zap70, cd8a, sla2*), a population of cytokine producing cells, likely representing T-cells (*il4, il13, il11, ca2, il34, il6*) as well as macrophages (*marco, ccl34a.4, mrc1b, c1qa/b/c, grn1*) and neutrophils (*cpa5, lect2l, mmp9, npsn, scpp8*). Additional cell types within the choroidal TME included fibroblasts (*col1a1a, col1a1b, col1a2, col5a1, dcn, serpinf1*), pericytes (*pdgfrb, notch3, foxf2b, cd248a*), Schwann cells (*dlx5a, apoda.1, mpz, plp1b, cd59, s100b, mbap*) and vasculature endothelial cells (*vwf, flt4, epas1b, ecscr, fabp11a*) (Figure 2D-F, **Table S1**).

**Figure 3:**
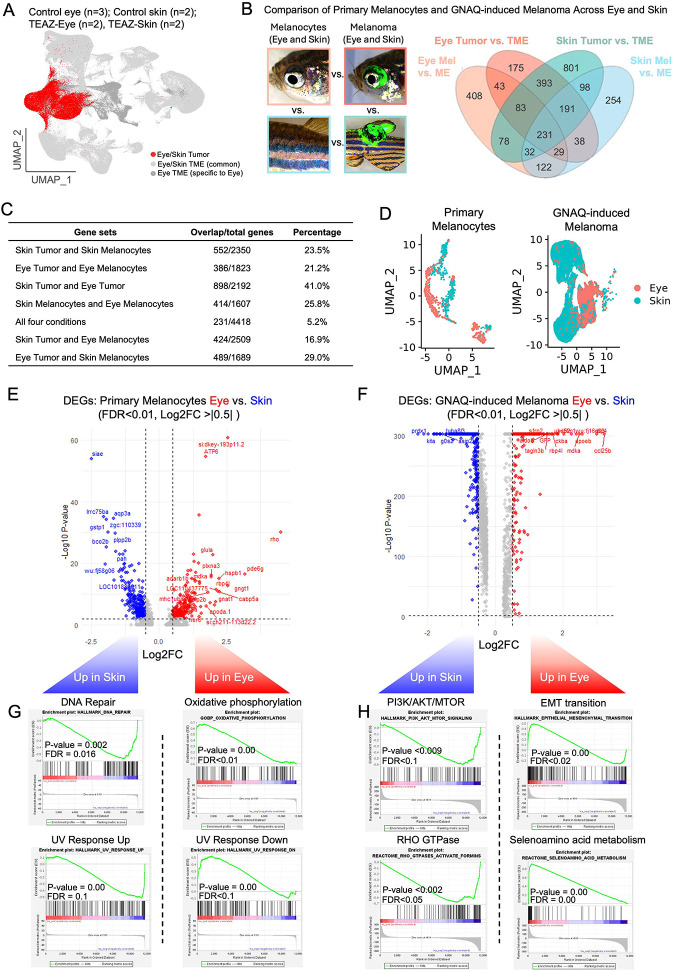
Comparison of GNAQ-driven tumors and primary melanocytes in the skin and eyes of adult zebrafish. (**A**) UMAP representation of GNAQ-positive tumors generated using TEAZ-Eye and TEAZ-Skin, along with their respective control tissues using scRNA-seq. Tumor samples include three dissociated wild-type eyes harboring UM tumors generated via TEAZ-Eye and two dissociated wild-type skin UM tumors generated via TEAZ-Skin (n=2 sequencing replicates). Control eye samples consist of a pool of two uninjected tumor paired wild-type eyes, three mock-injected sibling-matched wild-type eyes, and four uninjected sibling-matched wild-type eyes (n=3 sequencing replicates). Control skin samples consist of dissociated normal skin from two wild-type zebrafish (n=2 sequencing replicates). Cell clusters are annotated as tumor cells (red), tumor microenvironment (TME) cells shared by both eye and skin samples (light gray), and TME cells specific to the eye samples (dark gray). (**B**) Live imaging of representative samples used for single-cell RNA-Seq analysis and Venn diagram showing overlapping genes between TEAZ-Eye, TEAZ-Skin, choroidal melanocytes, and skin melanocytes compared to the cells in their respective microenvironments. Enriched genes for each condition were identified by differential expression analysis comparing melanocytes to the microenvironment (ME) or tumor cells to the tumor microenvironment (TME), using a threshold of log2FC > 0.5 and q-value < 0.01. (**C**) Table displaying overlaps between enriched gene sets from (B). Percentages represented as the number of overlapping genes over the total unique genes of both gene sets. (**D**) UMAP representation of primary melanocytes and UM tumor cells from both eye (salmon) and skin (teal). (**E**) Volcano plot of differentially expressed genes (DEGs) between eye and skin primary melanocytes (log2FC > ∣0.5∣ and q-value < 0.01). (**F**) Volcano plot of differentially expressed genes (DEGs) between eye and skin UM tumors (log2FC > ∣0.5∣ and q-value < 0.01). (**G**) GSEA enrichment plots of pathways that were enriched in skin melanocytes and of pathways enriched in eye melanocytes from (E). P-values and FDRs as labelled. (**H**) GSEA enrichment plots of pathways that were enriched in UM skin tumors and of pathways enriched in UM eye tumors from (F). P-values and FDRs as labelled..

We next compared choroidal melanocytes and UM tumors, revealing a significant overlap in gene expression (n=644; hypergeometric p < 0.0001) alongside a large set of tumor-enriched genes (n=1177) (Figure 2G). Shared genes included melanocytic regulators (*ednrb, sox10, foxd3, mitfa, mlpha, kita, mlana, pax7b*), supporting a melanocytic origin, and many of these transcripts were further upregulated in UM tumors compared to choroidal melanocytes (Figure 2H). HOMER promoter motif enrichment analysis of genes enriched in UM tumors revealed binding motifs for the MiT/TFE family of transcription factors, as well as PAX7 and HOXB13, suggesting that these regulators may contribute to UM-specific transcriptional programs (Figure 2G’) Differential expression analysis further identified several genes enriched in UM tumors and associated with patient survival, including *lgals2a, fabp3, gch2*, and the proto-oncogene *kita* (Figure 3H). High expression of *FABP3, FABP5, KIT, GCH,* and *LGALS2* correlated with worse overall survival (OS) and disease-free survival (DFS) in UM (Figure 3 **I-K**, Figure S7), and these genes were preferentially associated with TCGA molecular subtypes 3 and 4, representing the more aggressive forms of UM (Figure S8, **Table S2**).^55^

### Cancer associated fibroblasts within the uveal TME upregulate components of the extracellular matrix (ECM)

Recent studies have significantly advanced our understanding of the TME as an active driver of melanoma progression.^56,57^ While it is well established that components of the TME, particularly cancer-associated fibroblasts (CAFs), play a key role in malignancy,^58-60^ *in vivo* models to study such interactions in UM remain limited. Our model allows for direct comparison of the TME in TEAZ-Eye and control injected eyes within the same animals. We compared the transcriptome of CAFs and normal fibroblasts in wild-type zebrafish. Upon reclustering and differential gene expression analysis of normal eye fibroblasts and CAFs, we found that several ECM proteins were upregulated, indicating remodeling of the ECM in the presence of UM. Enriched genes broadly fell into four categories: ECM, ECM regulators, TNFs, and collagens. Notably, fibronectin (*fn1a*) was among the most significantly upregulated gene in CAFs compared to normal fibroblasts (q-value < 0.0001). Several collagens were also found to have higher expression in CAFs, including Collagen I (*col1a1b, col1a2),* Collagen V (*col5a1, col5a2a, col5a3a)* and Collagen XII (*col12a1a)* (Figure 2L, **Table S3**).

To control for injection-related fibrosis potentially causing fibroblast activation and upregulation of ECM components, we mock-injected three wild-type eyes with plasmids containing *mitfa*:GFP and *mitfa*:Cas9 cassettes followed by electroporation (mock-TEAZ) and performed scRNA-seq. Fibroblasts from mock-injected sibling control eyes (three pooled eyes), non-injected sibling control eyes (four pooled eyes), and tumor-matched control non-injected eyes (three pooled eyes) were equally distributed on the UMAP, with no alteration in *fn1a* or *lgals2a* expression or other changes to extracellular matrix (ECM) genes upon mock-TEAZ injection (Figure S5, Figure S9). These findings suggest that TEAZ-Eye tumor induction, and not the physical injection, in zebrafish triggers a CAF response that may be critical to melanoma progression.

### Skin and eye melanocytes, and their resulting tumors, have unique gene expression profiles

With the ability to induce genetically identical tumors in both the eye and the skin of adult zebrafish, we next asked whether tumors arising in these sites were transcriptionally distinct. Any such differences could be due to the relationship between the unique microenvironments of the skin and eye with their respective tumors or they could arise from inherent differences in the untransformed melanocytic cells that reside in the skin or eye. To this end, we generated tumors using TEAZ-Eye and TEAZ-Skin with strategy B plasmids in wild-type zebrafish and compared them with control eye and skin tissue, respectively, using scRNA-seq (Figure S5). UMAP analysis revealed that while the tumors and the majority of microenvironment clusters overlap, the eye tissue expectedly had unique cell types corresponding to retinal bipolar cells, glial cells, photoreceptors, and retinal interneurons (Figure 3A). To identify transcriptional differences between TEAZ-Eye and TEAZ-Skin tumors, we compared the gene expression profiles of each tumor type relative to the combined TME from both sites. A similar analysis was performed for eye and skin melanocytes relative to the combined uninjected microenvironment (Figure 3B, **Table S4**). Notably, the strongest overlap was observed between genes upregulated in skin tumors versus TME and those upregulated in eye tumors versus TME, with 41% of the 2,192 unique differentially expressed genes (DEGs) shared, highlighting the broad transcriptional programs activated by *GNAQ*^Q209L^ expression, *tp53* loss, and *ptena/b* loss (Figure 3C). We hypothesized that the DEGs shared by only the two tumors and not the primary melanocytes would represent mechanisms that are essential for UM growth and survival. In line with this prediction, IPA analysis of this gene subset revealed that pathways pertaining to cancer proliferation and metabolism were enriched (Figure S10A). Furthermore, we noticed an enrichment of WNT signaling, PTEN regulation, and RAF/MAPK pathways which have been previously implicated as important signaling cascades in UM.^61-69^ Interestingly, only 5% of DEGs overlapped across all datasets (eye and skin melanocytes and tumors), and we predicted these genes to be associated with melanocyte identity and survival (Figure 3C). IPA analysis confirmed this prediction with the most enriched pathway being MITF-M dependent gene expression. Other enriched terms were signaling events that are downstream of GPCRs, such as GNAQ, and pathways that are essential for survival such as nucleotide biosynthesis and amino acid regulation of mTORC1 (Figure S10B). Notably, only 25.8% of DEGs overlapped between eye and skin melanocytes, indicating substantial transcriptional differences in melanocytes originating from distinct anatomical regions (Figure 3C). We next asked whether transcriptional differences in primary melanocytes would be reflected in the corresponding tumors, that is, whether eye melanocytes would be more similar to eye tumors than skin melanocytes. Interestingly, the overlap of unique DEGs between eye melanocytes and eye tumors relative to their respective microenvironments (386/1823) was not significantly different than the overlap between skin melanocytes and eye tumors (489/1689) (permutation test, p-value = 1, 10,000 permutations) (Figure 3C). In contrast, the overlap of unique DEGs between skin melanocytes and skin tumors (552/2350) was significantly greater than the overlap between eye melanocytes and skin tumors (424/2509) (permutation test, p-value = 0, 10,000 permutations).

While this analysis allowed for consideration of genes that were differentially expressed in the same direction and magnitude versus the microenvironment, it would miss the genes that are differentially regulated between the two tissue types directly. Therefore, we reclustered just the normal melanocytes from both eye and skin for direct comparison and found that skin and eye melanocytes clustered separately on the UMAP (Figure 3D). Our analysis returned 186 DEGs enriched in eye melanocytes (log2FC > ∣0.5∣, q-value < 0.01) and 229 DEGs enriched in skin melanocytes (log2FC > ∣0.5∣, q-value < 0.01) with 3387 genes not differentially regulated (q-value > 0.01, expressed in at least 10% of cells), displaying modest transcriptional variance between melanocytes of the skin and the eye (Figure 3E, **Table S5**). GSEA analysis of these DEGs between eye and skin melanocytes revealed that skin melanocytes were enriched for DNA repair pathways (p-value < 0.002, FDR < 0.016) and for genes that are upregulated during UV response (p-value < 0.009, FDR < 0.081) (Figure 3G). On the other hand, DEGs in eye melanocytes showed an enrichment for the genes involved in downregulating the UV response pathway (p-value < 0.000, FDR < 0.090) and for the gene ontology oxidative phosphorylation pathway (p-value < 0.000, FDR < 0.006). We also reclustered and directly compared skin and eye tumors, with a similar tissue-specific grouping observed (Figure 3D). 230 DEGs were enriched in the eye tumors (log2FC > 0.5, q-value < 0.01) and 375 DEGs were enriched in the skin tumors (log2FC > 0.5, q-value < 0.01), while 5,754 genes remained not differentially regulated between the two tumor types (q-value > 0.01, expressed in at least 10% of cells) (Figure 3F). Interestingly, there was an enrichment of the epithelial to mesenchymal transition dataset (p-value < 0.000, FDR < 0.020) in the eye tumor as well as an enrichment for the metabolism of selenoamino acids (p-value < 0.000, FDR < 0.000) (Figure 3H). The skin tumor was instead enriched for PI3K-AKT-MTOR signaling (p-value < 0.009, FDR < 0.086) and pathways for RHO GTPases activating formins (p-value < 0.002, FDR < 0.058). Therefore, after both types of analyses we conclude that both the primary melanocytes and the GNAQ-driven melanomas are significantly different between the eye and the skin emphasizing the importance of studying UM, and melanocytes in general, in their proper anatomical context.

### Melanocyte progenitor cells are highly susceptible to oncogenic GNAQ transformation in *mitfa*-deficient zebrafish.

**Figure 4:**
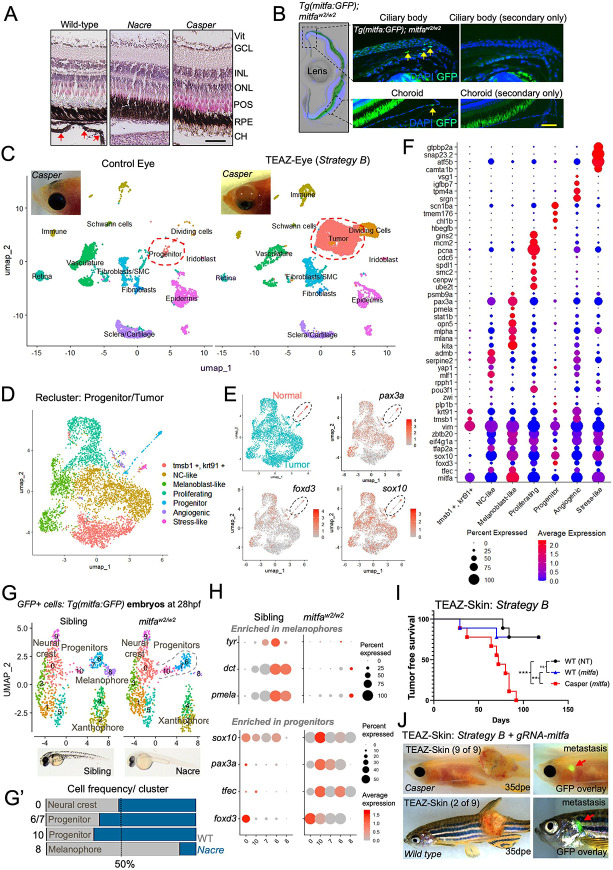
*mitf*-independent melanocyte progenitor cells are expanded in *mitfa*-deficient zebrafish and are susceptible to *GNAQ*^Q209L^ transformation. (**A**) H&E analysis representation of the retina and choroid in wild-type (left), *nacre* (middle), and *casper* (right) zebrafish (20x magnification). Note the loss of melanocytes (red arrows) in the choroid of *nacre* and *casper* eyes. “Vit” = vitreous, “GCL” = ganglion cell layer, “INL” = inner nuclear layer, “ONL” = outer nuclear layer, “POS” = photoreceptor outer segment, “RPE” = retinal pigmented epithelium, “CH” = choroid. (**B**) Immunofluorescent analysis of transgenic *Tg(mitfa:GFP); mitfa^w2/w2^* (*nacre*) zebrafish using an anti-GFP antibody. Sections were counterstained with DAPI to visualize nuclei. Representative images showing GFP-positive cells in the ciliary body and choroid are shown by yellow arrow heads. Secondary-only antibody was used as background control. (**C**) UMAP representation of single-cell RNA sequencing (scRNA-seq) data from three paired dissociated *casper* eyes and from three *casper* eyes harboring uveal melanoma (UM) tumors induced using Strategy B via TEAZ-Eye (n=1 sequencing replicate for each). Annotated cell clusters are labeled. Progenitor cells and UM tumor clusters are outlined with dashed red lines. Representative brightfield images of a normal eye and a UM tumor in *casper* zebrafish are shown in the top left corner of the UMAP. (**D**) Re-clustered UMAP representing progenitor cells from control eyes and uveal melanoma (UM) tumor cells from TEAZ-Eye–injected *casper* zebrafish. Heterogeneous cell populations as labeled. (**E**) Re-clustered UMAP from (D) showing melanocyte progenitor cells from control eyes in red and UM tumor cells from TEAZ-Eye in blue. Feature plots for *pax3a, foxd3* and *sox10* expression are shown, dashed line marks the progenitor cell population. (**F**) Dot plot illustrating differentiational expressed genes (log2FC > 0.5; q-value < 0.05) between heterogeneous cell populations in (D). Dot size indicates the percentage of cells expressing each gene; color intensity (blue to red) reflects normalized average expression levels (low to high). (**G, G’**) UMAP obtained after clustering GFP-positive cells sorted from *Tg(mitfa:GFP)* and *Tg(mitfa:GFP); mitfa^w2/w2^* (*nacre*) zebrafish embryos at 28 hours post fertilization (hpf). Annotated cell clusters as labelled. (**G’**) Comparison of relative cluster size for the two genotypes; *Tg(mitfa:GFP)* and *Tg(mitfa:GFP); mitfa^w2/w2^* (*nacre*) in (G). (**H**) Dot plot illustrating differentiational expressed genes (log2FC > 0.5; q-value < 0.05) in melanophores and progenitor cells in *mitfa^w2/w2^* and sibling embryos at 28hpf. Dot size indicates the percentage of cells expressing each gene; color intensity (grey to red) reflects normalized average expression levels (low to high). (**I**) Kaplan–Meier curves comparing tumor-free survival in wild-type and *casper* zebrafish following TEAZ-Eye injection using Strategy B plasmids for *mitfa* conditional knockout. Wild-type zebrafish were injected with either Strategy B plasmids plus non-targeting gRNAs (n=9) or Strategy B plasmids plus *mitfa*-targeting gRNAs (n=9). *Casper* zebrafish were injected with Strategy B plasmids and *mitfa*-targeting gRNAs (n=9). (**J**) Representative brightfield and GFP-overlay images of tumors that formed in *casper* (9 of 9) and wild-type zebrafish injected with *mitfa*-targeting gRNAs (2 of 9). Metastatic cells are indicated by red arrows.

H&E analysis of *nacre* and *casper* zebrafish eyes showed loss of choroidal melanocytes (Figure 4A), whereas melanin in the RPE remains less affected, as previously demonstrated.^33^ These observations imply that an *mitfa*-independent melanocyte progenitor cell persists in *nacre* and *casper* eyes and that they are susceptible to oncogenic GNAQ transformation. To identify such cells, we crossed *nacre* (*mitfa*^*w2/w2*^) with transgenic *Tg(mitfa:GFP)* lines to create *Tg(mitfa:GFP); mitfa*^*w2/w2*^ zebrafish. We then harvested adult eyes at 5 months and performed anti-GFP immunofluorescence analysis, with the assumption that loss of *mitfa* does not alter expression of the *mitfa* promoter. While GFP+ melanocytes were readily detected in the choroid of *Tg(mitfa:GFP)* zebrafish (Figure 1F, Figure S3), staining of *Tg(mitfa:GFP); mitfa*^*w2/w2*^ eyes revealed rare cells with GFP-positivity (Figure 4B). Such GFP+ cells were primarily located in the ciliary body, with isolated cells also found in the choroid, suggesting the persistence of a *mitfa*-independent progenitor population in adult *mitfa*-deficient zebrafish (Figure 4B). We next asked whether such cells retain the ability to undergo melanocyte differentiation by using TEAZ-Eye to deliver plasmids containing the *mitfa*-promoter driving expression of *mitfa* (i.e., to overexpress *mitfa* mRNA). Interestingly, overexpression of *mitfa* transcript levels resulted in expansion of choroidal melanocytes in wild-type animals and the rescue of choroidal melanocytes in *mitfa*-deficient (*nacre*) zebrafish as shown by H&E analysis (Figure S11A-B’). To assess whether GFP-positive cells are sensitive to oncogenic transformation by GNAQ, we expressed *mitfa*:*GNAQ*^*Q209L*^ in *Tg(mitfa:GFP); mitfa*^*w2/w2*^ zebrafish using TEAZ-Eye. In all injected fish (n=3), UM tumors were GFP-positive, suggesting they originated from GFP+ progenitor cells within the eye of *Tg(mitfa:GFP); mitfa*^*w2/w2*^ zebrafish (Figure S11C).

### Melanocyte progenitor cells express neural crest marker genes in *mitfa*-deficient zebrafish.

We performed scRNA-seq and pseudotime analysis on 3 pooled UM tumors from the eyes of *casper* zebrafish and compared them to the pooled tissue from 3 normal *casper* eyes (Figure 4C, Figure S12). Within the *casper* eyes, we identified a population of cells expressing the *nacre* allele that clustered closely with UM tumors under TEAZ-Eye conditions, termed progenitors (Figure S13A-B **and Tables S6-8**). We re-clustered the progenitor and tumor cells (Figure 4D), revealing seven distinct clusters consisting of neural crest-like (NC-like), melanoblast-like, proliferating, progenitor, angiogenic, stress-like, as well as an unidentified cluster marked by *tmsb1, krt91* and *vim* (Figure 4D, **Table S9**). Unsupervised pseudotime analysis supports a lineage trajectory from the progenitor cells to heterogeneous UM tumor cells (Figure S13C). Notably, the progenitor cell population in normal *casper* eyes expressed neural crest and melanocyte stem cell marker genes, including *tfap2a, foxd3, sox10*, *pax3a, vim,* and *yap1* (Figure 4E-F). UM tumor cells retained expression of these genes but activated additional genes associated with pigment progenitor cell function, including *tfec* and *kita* (Figure 4F). Notably, *mitfa* expression in tumors and progenitor cells in *casper* zebrafish reflects the expression of the mutant *mitfa*^*w2/w2*^ allele, which functions as a lineage tracer to mark cells that would normally express *mitfa* under wild-type conditions (Figure S13B). Interestingly, despite the lack of Mitfa activity in *casper* zebrafish, GNAQ-positive UM tumors included a melanoblast-like cluster that exhibited expression of pigmentation genes, including *pmela, mlpha*, and *mlana,* likely explaining the occasional emergence of melanin within tumors as they progress in *casper* zebrafish.

### *mitfa*-deficient zebrafish exhibit expanded *pax3a* and *tfec* positive melanocyte progenitors during embryogenesis.

Having established that an Mitfa-independent progenitor population gives rise to UM in *mitfa*-deficient zebrafish, we next examined whether this population is expanded relative to wild-type zebrafish. To test this prediction, we performed scRNA-seq on GFP-positive cells sorted from *Tg(mitfa:GFP);mitfa*^*w2/w2*^ and *mitfa*^*+/w2*^ sibling-matched transgenic zebrafish embryos at 28 hours post fertilization (hpf), when melanocytes (zebrafish melanophores) begin to differentiate (Figure 4G, Figure S12). We assigned cell-type annotations based on our previously annotated GFP-positive cells from *Tg(mitfa:GFP)* embryos.^30^ The 10 clusters included six main cell types: neural crest cells (*sox10, foxd3*), a tripotent precursor of melanoblasts (M), iridoblasts (I), and xanthoblasts (X) (*tfap2a*, *cdkn1ca, slc15a2, ino80e, id3, mycn, tfec*), termed MIX cells, a cluster that expressed high levels of melanoblast/xanthoblast markers (*mitfa, erbb3b, impdh1b, gch2, id3*), termed MX cells, a melanoblast cluster (*mitfa, dct, pmel, tyr*), as well as two additional clusters corresponding to xanthoblasts and xanthophores (**Table S10**). For this analysis we referred to MIX and MX cells as melanophore progenitors (Figure 4G). As expected, melanophore cells were reduced in *Tg(mitfa:GFP): mitfa*^*w2/w2*^ zebrafish (Figure 4G-G’). However, melanophore progenitors were expanded in *Tg(mitfa:GFP); mitfa*^*w2/w2*^, while neural crest cells remained unaffected (Figure 4G-G’). Melanophore markers were downregulated in GFP-positive cells sorted from *Tg(mitfa:GFP); mitfa*^*w2/w2*^, whereas melanocyte progenitor markers (*pax3a, sox10, foxd3*), as we identified in adult zebrafish eyes, were upregulated in embryonic progenitors from *Tg(mitfa:GFP); mitfa*^*w2/w2*^ embryos (Figure 4H, **Table S11**). A role for *pax3a* in adult fish eye tissue had not been previously characterized; however, recent studies have identified PAX3 expression in ocular surface melanocytes,^70^ and another study reports an association between PAX3 and stem cell markers in UM progression.^71^ Consistent with these observations, we identified a *pax3* expressing cell population in adult C57BL/6 mouse eyes in the limbal region, and rarely in the choroid (Figure S14). These findings support a rare *pax3*-expressing population in both zebrafish and mouse eyes and may mirror *pax3* expression in the melanocyte stem cell in the skin.^72,73^

### Conditional deletion of *mitfa* in adult zebrafish fails to recapitulate germline loss-of-function effects on tumor-free survival in GNAQ-driven melanoma.

We next investigated whether the accelerated growth of GNAQ-driven tumors in *mitfa*-deficient zebrafish, compared to wild-type animals, was due to an expansion of progenitor cells or the cell-intrinsic loss of Mitfa activity. To this end, we performed conditional *mitfa* knockout experiments using Strategy B plasmids in combination with either (1) non-targeting control gRNAs or (2) gRNAs targeting exon 5 of *mitfa*. TEAZ-Skin was performed in three experimental groups: (1) *casper* zebrafish (n=9) with gRNAs targeting *mitfa* (which controls for off-target effects of the gRNA and for potential function of the *mitfa*^*w2/w2*^ allele), (2) wild-type zebrafish (n=9) with non-targeting (NT) gRNAs, and (3) wild-type zebrafish (n=9) with *U6*:gRNA-*mitfa*. GFP expression was confirmed in all injected zebrafish (**Table S12**). Within 100 days post-electroporation (dpe), tumors developed in 9/9 casper, 2/9 wild-type (+U6:gRNA-mitfa), and 2/9 wild-type (+U6:gRNA-NT) zebrafish (Figure 4I-J). Of note, *mitfa*-deficient tumors that formed in wild-type or *casper* zebrafish developed distal metastases, an observation not seen in *mitfa* wild-type TEAZ-Skin injected zebrafish (Figure 4J, Figure S15). Our findings support a model in which failed differentiation as well as the expansion of melanocyte progenitor cells in *mitfa*-deficient animals creates a permissive cellular environment for GNAQ-induced transformation and reduced tumor latency.

### Transcriptional programs enriched in BRAF- versus GNAQ-driven melanoma overlap with specific melanocyte progenitor populations.

**Figure 5:**
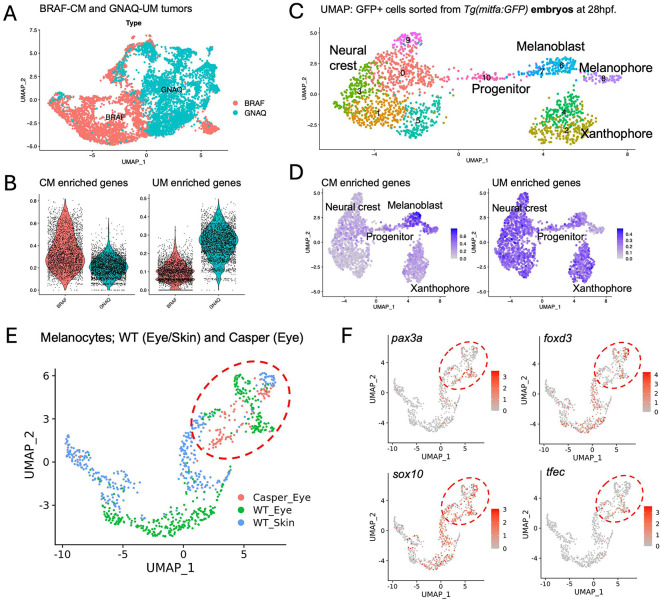
Oncogenic GNAQ and BRAF transform transcriptionally distinct cells within the melanocyte lineage. (**A**) UMAP obtained after clustering tumor cells from *Tg(mitfa:BRAF^V600E^): tp53^−/−^* : *mitfa^w2/w2^* zebrafish where TEAZ-Skin was used to rescue *mitfa* expression and knockout *ptena* and *ptenb* to induce cutaneous melanoma^32^, along with our GNAQ-induced tumors by TEAZ-Skin (Strategy B: *mitfa:Cas9, U6:*gRNA-*tp53, U6:*gRNA-*ptena, U6:*gRNA-*ptenb, mitfa:GNAQ*^Q209L^-*mitfa:GFP*). BRAF-induced tumors in red and GNAQ-induced tumors in blue. (**B**) Violin plot showing differentially expressed genes (log2FC > 0.5, q-value < 0.05) between BRAF-induced and GNAQ-induced tumors from (A). The plot displays the distribution of gene expression across individual cells, with the width indicating cell density at specific expression levels and the height representing the range of expression values. (**C**) Integrated UMAP after clustering GFP-positive cells sorted from *Tg(mitfa:GFP)* and *Tg(mitfa:GFP); mitfa^w2/w2^* (*nacre*) zebrafish embryos at 28 hours post fertilization (hpf). Annotated cell clusters as labelled. (**D**) Average enrichment of signature genes from BRAF- and GNAQ-driven tumors projected onto the UMAP in (C) using UCell with 'FeaturePlot' function. Signature genes represent the top 50 enriched genes from each tumor genotype that are also expressed in the embryonic melanocyte lineage. (**E**) UMAP of primary melanocytes and melanocyte progenitors from 9 control wild-type eyes, 2 control wild-type skin sections, and 3 control *casper* eyes. Cells are labeled according to their genotype (*casper* and wild-type) and tissue origin (eye and skin). (**F**) Feature plots representing the relative mRNA expression of *pax3a, foxd3, sox10*, and *tfec* in melanocytes described in (E). Color intensity (grey to red) reflects normalized average expression levels (low to high). The red dotted line indicates cells that are clustering closely with the melanocyte progenitors identified in *casper* eyes.

Oncogenic BRAF and NRAS are unable to promote transformation in the absence of *mitfa,*^29,32^ suggesting that distinct transcriptional programs, potentially reflecting unique cellular origins, are required for UM and CM transformation. To address this hypothesis, we analyzed previously published scRNA-seq datasets in transgenic *Tg(mitfa:BRAF*^*V600E*^*): tp53*^*−/−*^ : *mitfa*^*w2/w2*^ zebrafish where TEAZ-Skin was used to rescue *mitfa* and induce melanoma,^32^ to our GNAQ-induced tumors by TEAZ-Skin (Figure 5A). We predicted that by analyzing primary tumors, the transcriptional signature of the origin cell will be retained and can be identified by overlapping gene signatures identified in the embryonic melanocyte lineage.^30^ Interestingly, while the expression of *mitfa* was significantly higher in GNAQ-positive tumors, the expression of pigmentation genes; including *tyrp1b, dct, scl45a2, sox10* and *pmela* were significantly higher in BRAF-positive melanoma (Figure S16). Genes enriched in GNAQ-UM and BRAF-CM were identified by those with a Log2FC > 1 and Adj p-value < 0.0001 between the two tumor types (Figure 5B, **Table S13**). We next overlapped this differential gene signature with genes expressed by cells within the melanocyte lineage isolated from zebrafish embryos (Figure 5C). As expected, BRAF-driven tumors strongly mapped to the melanoblast and melanophore clusters (Figure 5D), supporting recent findings that BRAF-driven melanoma originates from melanoblasts, a melanocyte progenitor population^32,74^. When comparing GNAQ-enriched genes to the melanocyte lineage, we found that the signatures were not confined to a single cluster but were distributed among neural crest cells, melanocyte progenitors, and xanthophore clusters (Figure 5D; Figure S17). Such results support the hypothesis that GNAQ- and BRAF-driven melanomas depend on distinct transcriptional programs, reflecting less and more differentiated melanocytic states, respectively.

We next asked whether less differentiated melanocytes, similar to the *mitfa*-deficient progenitors in *casper* eyes, are also present in adult wild-type eyes. Since melanocyte stem cells are known to reside in the zebrafish skin,^28^ we included skin tissue as a control and reclustered melanocytes from wild-type skin and eyes with progenitor cells from *casper* eyes. Interestingly, a subset of wild-type melanocytes from both eye and skin samples clustered closely with *casper* progenitors (Figure 5E, Figure S18) and expressed pigment cell progenitor marker genes, including *pax3a, sox10, foxd3,* and *tfec* (Figure 5F). These findings suggest either plasticity among melanocytes within the eye or the existence of an eye melanocyte stem cell population in adult wild-type zebrafish. Moreover, such cells may be more susceptible to transformation by *GNAQ*^Q209L^ and could represent the cells of origin for UM tumors in this context.

## Discussion

Herein, we present an immunocompetent zebrafish model of primary UM where tumors arise within the choroid and progressively invade surrounding ocular structures. This model enables the study of the earliest events of UM pathogenesis and how anatomical context impacts disease progression. Notably, genes identified through our analysis correlate with survival differences in human UM, highlighting the model’s potential for mechanistic studies on gene function in UM pathogenesis. Compared to transgenic models, our system produces anatomically correct tumors at an accelerated rate, offering an efficient platform for studying UM progression and therapeutic responses.

By generating UM tumors from the same anatomical location as the majority of human UM, we were able to conduct key analyses such as the comparison of the tumor to untransformed primary choroidal melanocytes and the tumor microenvironment. This method allowed for the identification of genes that are upregulated in the tumor and therefore important for disease onset and progression. Specifically, we found that fatty acid binding proteins, particularly *fabp3*, are enriched in UM tumors. While lipid droplets have been identified as vulnerable targets in CM and regulators of melanoma plasticity,^32,75^ lipid signaling in UM has received limited attention.^76-79^ Interestingly, high expression of *FABP3* as well as *FABP5* associated with worse disease-free survival in patients with UM but not in CM. Interestingly, our analysis also identified *LGALS2* and *GCH*, two genes whose expression was strongly associated with worse overall survival in UM but intriguingly showed the opposite in CM. Finally, we found greater *kita* expression in GNAQ-driven tumors compared to choroidal melanocytes, a result that is particularly notable given recent studies linking KIT-positive UM tumors with poor prognosis and monosomy of chromosome 3.^80^ Furthermore, expression of these genes was highest in TCGA molecular classes 3 and 4 of the UM dataset, the subset of patients with the highest rates of metastatic incidence and BAP1 loss.^55^ Such observations show that this model can uncover genes that are likely to have key, disease-specific roles in UM whose function could be further explored through genetic perturbations in zebrafish. These genes could also be developed into new biomarkers of disease risk to refine the identification of patients likely to progress to metastatic disease.

Although UM and CM both arise from cells within the melanocyte lineage, they differ significantly in their biology and clinical outcomes. To explore how anatomical context influences transcriptional states, we compared genetically identical tumors derived in the skin and eye and found that eye-derived tumors display distinct expression profiles. To explore whether the differences in tumors were due to inherent differences in their primary cells, we also compared normal melanocytes from the eye and the skin. While we did find some transcriptional distinctions between eye and skin melanocytes, these variances only represented a small fraction of the differentially expressed genes between eye and skin melanomas. These results suggest that the transcriptional profiles of genetically identical tumors in distinct anatomical locations are influenced by more than the intrinsic differences in their origin cells, perhaps varying due to signals from their unique microenvironments. Of the expression differences between normal eye and skin melanocytes, GSEA pathway analysis revealed that skin melanocytes are enriched for UV responses and DNA damage repair. Zebrafish, like humans, have been shown to alter their melanin production in response to UV exposure.^81^ Our results suggest that skin melanocytes in the zebrafish may be more primed to enact UV-protective functions than eye melanocytes, even with no UV exposure. Furthermore, the enrichment of oxidative phosphorylation in eye melanocytes and of selenoamino acid metabolism in eye tumors suggests metabolic differences between melanocytes derived from the eye or skin. Interestingly, disruptions in selenoamino acid metabolism have been shown to adjust the TME and growth of primary tumors in a number of cancers, including melanoma.^82^ Pathway analysis between skin and eye tumors has also revealed potential differences in the progression of these tumors. Eye tumors were enriched for EMT genetic programs, suggesting that eye melanocytes may exist in a more mesenchymal state than skin melanocytes or that greater expression of EMT programs is necessary for metastatic spread out of the eye and towards distant sites like the liver. On the other hand, skin tumors were enriched for the certain arms of the GNAQ signaling cascade,^5,11-13^ namely PI3K/AKT/MTOR signaling and RHO GTPases activating formins. Further investigation is needed to confirm whether microenvironment differences or anatomical origin of melanocytes can influence the relative importance of oncogenic pathways in the setting of *GNAQ*^Q209L^ expression. Using our animal model to compare genetically identical tumors, and their primary cells, across different anatomical sites has revealed distinct biological behaviors, highlighting the model's potential for further investigations into site-specific tumor biology and therapeutic strategies.

An interesting distinction between GNAQ and BRAF oncogenes is their differential dependency on Mitfa activity for tumorigenesis. While BRAF^V600E^ requires Mitfa activity to induce melanoma,^27,29^ GNAQ^Q209L^-driven tumor latency was significantly reduced in *mitfa*-deficient zebrafish compared to wild-type controls. Our results are consistent with prior studies showing that *mitfa*-deficient zebrafish activate distinct signaling pathways, favoring YAP over MAPK signaling, and give rise to tumors with reduced latency compared to wild type tumors. Moreover, manipulating YAP activity in wild-type animals was shown to recapitulate the enhanced growth seen in *mitfa*-deficient tumors.^40^ We observed high *yap1* expression in melanocyte progenitor cells of *mitfa*-deficient zebrafish, suggesting that elevated Yap1 activity may be carried over from the progenitor cell during transformation. While germline *mitfa* loss accelerates tumor onset, our data suggest this effect is not solely due to the deletion of Mitfa from differentiated melanocytes. Instead, we propose that germline *mitfa* deficiency expands a population of *pax3a*- and *tfec*-positive melanocyte progenitor cells, which are more susceptible to GNAQ-induced transformation. Notably, conditional loss of *mitfa* in differentiated melanocytes may activate progenitor-like transcriptional programs, increasing their vulnerability to oncogenic transformation. Alternatively, *mitfa* loss may impair the differentiation of melanocyte progenitors, preventing the formation of GNAQ-positive nevi and promoting direct progression to malignancy. Interestingly, these progenitor cells appear to maintain a neural crest signature, as evidenced by the relatively high expression of additional markers such as *foxd3, pax3a, sox10,* and *tfec*. ^30,31,83-85^ Together, these findings suggest that germline loss of *mitfa* accelerates GNAQ-driven transformation of melanocyte progenitor cells by promoting their expansion and maintaining a neural crest-like transcriptional profile. Identifying the transcriptional and signaling mechanisms within *mitfa*-independent melanocyte progenitor cells that facilitate UM onset and progression will have significant clinical implications, particularly for the differential diagnosis of high-risk lesions and the rational design of targeted therapies.

Studies in human UM have shown that less pigmented tumor regions are more metastatic and that BAP1-negative tumors exhibit a stem-like phenotype.^1,2,86,87^ Our findings raise intriguing questions about whether the progenitor cells observed in *mitfa*-deficient zebrafish are similarly predisposed to a more undifferentiated, stem-like phenotype, contributing to their enhanced transformation potential and aggressive tumor behavior. Interestingly, the direct comparison of melanocytes and tumors from the eye or skin against their microenvironments revealed that skin melanocytes overlap with the skin tumor greater than the overlap of eye melanocytes with the skin tumor. However, this was not true in reverse with eye melanocytes being no more similar to the eye tumor than skin melanocytes are. These findings suggest that eye tumors may originate from a less differentiated cell compared to the melanocytic cell type that CM arises from. This progenitor cell may be underrepresented in the normal eye melanocyte population, leading to the observed lower transcriptional overlap between total enriched genes in eye tumors and normal eye melanocytes. Furthermore, re-clustering of normal WT melanocytes and melanocyte progenitor cells from *mitfa*-deficient zebrafish demonstrates that both the eye and skin have melanocyte populations that cluster closely with melanocyte progenitors. These melanocyte populations may be less differentiated as evidenced by increased expression of *pax3a*, *tfec*, and *foxd3*. Whether this subset of melanocytes represents bona fide melanocyte stem cells or melanocytes that can oscillate between differentiation states within the eye remains to be uncovered.

Furthermore, differences in *mitfa* dependency between BRAF-driven CM and GNAQ-driven UM suggest fundamental distinctions in their cellular origins. Although MITF-independent melanocytic cells are unlikely to be the cellular origin of CM in zebrafish models, studies have shown that MITF-independent BRAF-positive melanoma cells are central to disease recurrence^45^. Our results highlight that *mitfa* expression does not directly reflect Mitfa activity, as *mitfa*-target genes are more highly expressed in BRAF-positive melanoma despite lower *mitfa* expression compared to GNAQ-positive melanoma. GNAQ-driven transformation may be facilitated by Mitfa paralogs such as Tfec and Tfeb. The Mitfa paralog Tfec has been shown to activate pigmentation genes in both the retinal pigment epithelium and melanocyte progenitor cells. Tfec can also rescue ectopic melanocytes in *nacre* zebrafish embryos.^31,33^ Moreover, we have recently shown that the MITF paralog TFE3 promotes cellular plasticity and stemness genes in MITF-low melanoma cells,^88^ suggesting that MITF paralogs are compelling candidate transcription factors in UM pathogenesis.

Although understudied, the choroidal stroma is abundant in fibroblasts that produce various ECM proteins, including collagen, elastin, laminin, fibrin and fibronectin.^89-92^ This microenvironment differs from that of the keratinocyte-rich epidermis where CM arises. The differences in TME between UM and CM are likely to play an important role in the phenotypic differences observed in these tumors. Recent studies show that increased fibronectin promotes a less differentiated and more mobile phenotype in murine melanocytes *in vitro*.^93^ Our finding that fibronectin (*fn1a* and *fn1b)* was upregulated in CAFs compared to normal fibroblasts provides evidence that fibronectin may promote invasiveness or dedifferentiation in a malignant, *in vivo* context. Similar to other studies finding an accumulation of collagen I in the TME of various cancer types, we found upregulation of *col1a1b* and *col1a2* in CAFs relative to normal fibroblasts, indicating increased collagen I deposition.^59,60^ The precise mechanism of how different subclasses of CAFs regulate UM differentiation state and propensity to metastasize through ECM remodeling is still to be defined.

Another explanation for decreased tumor latency in *casper* zebrafish is that the global loss of *mitfa* could have secondary effects on other cell types, such as immune cells, which may influence tumor behavior. Furthermore, the presence of xanthophore, iridophore, and melanophore markers within UM tumor cells raises the possibility of a pluripotent progenitor population. This model also enables us to examine whether differences in differentiation potential contribute to tumor aggressiveness, as *casper* fish may exhibit more pronounced tumor acceleration due to the simultaneous blockade of two chromatophore differentiation pathways compared to single-pathway inhibition in *nacre* fish.

Our findings open numerous avenues for future research. This model can be used to study the effects of *BAP1* loss and other secondary mutations commonly associated with UM, such as *EIF1AX* and *SF3B1*. It also provides a platform for investigating metastatic events, as tumors emerge in defined primary sites. Establishing zebrafish UM cell lines from different anatomical locations and genetic backgrounds could offer further insights into the role of microenvironment and *mitfa* dependency in UM progression. Additionally, characterizing the melanocyte progenitor population in the eye and determining if a similar population exists in mammals could provide critical insights into uveal stem cell biology. While we identified a population of Pax3-positive cells in the mouse limbus, it remains to be determined if these cells are sensitive to oncogenic GNAQ transformation.

## Limitation of study

The use of *tp53* and *ptena/b* mutations in our UM model does not fully recapitulate the genetics of human UM. Mutations in *TP53* and *PTEN* are rarely observed in UM, where loss-of-function mutations in *BAP1* represent the most frequent tumor suppressor alteration.^67,94,95^ However, both *TP53* and *PTEN* signaling pathways have been shown to be altered in UM pathogenesis. *MDM2* overexpression, an upstream negative regulator of p53, and downregulation of *PERP*, a downstream effector of p53, have been demonstrated in many UM patients and are associated with worse outcome.^96-100^ Studies have also shown that over half of UM tumors have decreased PTEN immunostaining and its loss is correlated with decreased survival.^67-69^ Nevertheless, whether genetic knockout of these tumor suppressors recapitulate the dysregulation of these pathways in human UM remains an open question. Another limitation to our approach when examining the differences between genetically identical skin and eye-derived tumors is the inability to distinguish between purely microenvironmental effects and differences in cell of origin (melanocytes in the skin versus in the eye). To address this question, a syngeneic or immunodeficient model could be applied to transplant cells from a primary UM tumor into both anatomical sites. Additionally, our use of the *mitfa* promoter to drive oncogene expression presents a challenge in defining the exact cell of origin, as this promoter is active in both melanocyte progenitors and differentiated melanocytes. As such, it remains unclear whether tumors in *mitfa* wild-type fish (or those with conditional *mitfa* deletion) arise from a progenitor or a mature melanocyte. Lineage tracing studies will be essential to determine whether differentiated melanocytes possess the capacity to initiate primary UM in *mitfa*-competent or conditional *mitfa*-KO zebrafish.

## Resource availability

### Lead contact

Requests for further information and resources should be directed to, and will be fulfilled by, the lead contact, Colin Kenny, PhD (colin-kenny@uiowa.edu).

### Materials availability

Plasmid constructs and zebrafish generated in this study may be obtained by a request to the lead contact.

### Data and code availability

Datasets generated in this article are available from NCBI GEO accession number GEO: GSE307227. This study does not report original code. Any additional information required to reanalyze the data reported in this work paper is available from the lead contact upon request.

## Methods

### Experimental model and study participant details

#### Zebrafish husbandry

All zebrafish used in this study were bred and maintained at the University of Iowa Animal Care Facility. The zebrafish are kept consistently at 28°C, 7.4 pH, and controlled salt concentrations and cyclically receive 14 h of light followed by 10 hours of darkness. All zebrafish experiments were performed in compliance with the ethical regulations of the Institutional Animal Care and Use Committee at the University of Iowa and in compliance with NIH guidelines (protocol #3022523). Zebrafish embryos were maintained at 28.5°C and staged by hours or days post-fertilization (hpf or dpf).

#### Zebrafish mutant lines

Wild-type zebrafish in this study include the *AB* line and the *WIK* line. Transgenic lines include *casper* (*mitfa*^w2/w2^; *mpv17*^−/−^)^46,47^, *nacre* (*mitfa*^w2/w2^)^43^, *Tg*(*mitfa*:GFP)^18^, and *Tg*(*mitfa*:GFP); *mitfa*^w2/w2^. TEAZ-Eye and TEAZ-Skin was performed on adult zebrafish (greater than 4 months postfertilization).

#### Cloning

The MiniCoopR^29^ vector (MiniCoopR 2x*U6*:gRNA, *mitfa*:Cas9 was a gift from Leonard Zon Addgene plasmid # 118844) was modified to replace the *mitfa* coding sequence with *GNAQ*^Q209L^ by removing *mitfa* using StuI and SmaI restriction enzymes (NEB). Gibson assembly was used to insert *GNAQ*^Q209L^:T2A:GFP DNA as a gBlock hifi (IDT) with sequence on the 3’ (GAAGCTAACACATAGTTGAAC) and 5’ (CAATGCCAACTAAATTTCATG) as overhangs. Gibson assemblies were transformed using NEB 5-alpha competent *E. coli* cells via heat shock. *GNAQ*^Q209L^:T2A:GFP insertion was confirmed using Sanger sequencing (Primers: CAAGGAAGCCCGGCGGATCAACGA, CATACTTGTATGGGATCTTGAGTGTGTCCA, GTGGAGTCAGACAATGAGAACCGAATGGAG, CAAGCTGGAGTACAACTACAA).

*mitfa* sgRNA was designed using IDT’s CRISPR-Cas9 guide RNA design tool (sequence: ATGGACAAAGCTGGACCATG). *U6*:sgRNA-*ptena* was linearized by PCR (forward primer: GTTTAAGAGCTATGCTGGAAACAGCAT, reverse primer: GAACAAAGAGCTGGAGGGAG). Linear plasmid was purified from a 2% agarose gel using the Monarch GNA Gel Extraction Kit (#T1020S). ssDNA sequences for sgRNA-*mitfa* were annealed together in NE buffer r2.1. Gibson assembly was used to insert sgRNA-*mitfa* into linearized U6 plasmid. Assembled product was transformed into NEB 5-alpha competent *E. coli* cells via heat shock and selected with 50 ug/mL streptomycin. Insertion of sgRNA-*mitfa* was confirmed using PCR amplification of lysed bacteria from single colonies (forward primer: ATGGACAAAGCTGGACCATG, reverse primer: CTTAGCTGGATAACGCCAC). Positive products were isolated using the QIAGEN Plasmid Maxi Kit. Entire plasmid was sequenced using Plasmidsaurus sequencing service.

#### Transgene Electroporation of Adult Zebrafish Skin (TEAZ-Skin)

Adult zebrafish were anesthetized in 0.2% Tricaine prior to upright placement on a prewetted Kimwipe. Each injection consisted of 1000 ng total DNA: 100 ng of Tol2 plasmid and an equal division of the remaining 900 ng for the remaining constructs. Injection stocks contained a small volume (<1uL, 1:50 dilution) of green food coloring to visualize localization of injection. Injection volumes ranged from 1 to 1.5 uL depending on the injection mix, keeping 1000 ng total constant. Needles were pulled from 10 cm long filamented borosilicate glass capillaries at a pressure of 500, heat of 650, pull strength of 100, velocity of 200, and time delay of 40 in a Model P-97 Flaming/Brown micropipette puller from Sutter Instrument Co. Following injection, zebrafish were immediately electroporated (<1 min) by placing the cathode probe of ultrasound gel-coated Platnium Tweezertrode, 3MM, on the side of the injection site and the anode probe on the other side of the fish body. Electroporation was achieved with 5 60-ms pulses of 40 volts with a 1s pulse interval from a ECM 830 Electro Square Porator from BTX Harvard Apparatus. Following electroporation, zebrafish are placed into a recovery tank with fresh, gently stirred system water and are monitored until normal swimming resumes. Electroporated zebrafish are imaged weekly. *mitfa*:*GNAQ*^Q209L^; *mitfa*:GFP plasmid constructs were kindly provided by Dr. Jacqueline A. Lees (Massachusetts Institute of Technology, Cambridge, USA). *mitfa*:Cas9, *mitfa:*tdTomato, PCS2FA Tol2, *U6*:gRNA-*tp53, U6*:gRNA-*ptena,* and *U6*:gRNA-*ptenb* plasmids were kindly provided by Dr. Richard White (Sloan Kettering Institute, New York, USA).

#### Transgene Electroporation of Adult Zebrafish Eye (TEAZ-Eye)

Pre-injection preparation for eye injections is the same as for skin injections including: 0.2% Tricaine anesthesia, needle size, and injection concentrations. To inject the eye, a blunt spherical head of a pin was gently pressed against the bottom half of the zebrafish eyeball to reveal the dorsal posterior regions of the eye. The needle was gently pressed into this location, where the cornea is softer, just through the sclera, keeping the needle as superficial as possible as to not damage the back of the eye. Injection volumes were around 0.25 uL and were terminated with a slight visual swelling of the eye. Following injection zebrafish were immediately electroporated (<1 min) by the cathode probe of ultrasound gel-coated Platnium Tweezertrode, 1MM, on the superior side of the eye nearest the injection and the anode probe on the inferior side of the eye. Electroporation was achieved with 3 50-ms pulses of 75 volts with a 1s pulse interval from a ECM 830 Electro Square Porator from BTX Harvard Apparatus. Recovery and imaging procedures for these fish did not differ from the skin injections.

#### Imaging and image processing

Injected zebrafish were imaged using an upright Leica M205 FCA microscope with brightfield and GFP filters. Zebrafish were briefly anesthetized with 0.2% Tricaine and positioned on a Kimwipe mound for upright orientation. Images were taken and processed through the LAS X software. All histology slides were imaged using brightfield, DAPI, and GFP filters on an EVOS M5000 microscope from Invitrogen for individual images or on a Leica DMi8 microscope and stitched together by LAS X Thunder Imaging software for full tissue images.

#### H&E and IF – zebrafish tissue

Whole zebrafish or zebrafish eyes were fixed in 4% paraformaldehyde for 48 hours at 4°C and paraffin embedded in the University of Iowa Pathology Core. Fish were sectioned at 7 μM thickness and placed on Superfrost Plus Microscope slides from Fisher Scientific, baked at 60°C, stained with hematoxylin and eosin, and mounted with Cytoseal 60 from Epredia. For immunofluorescence, sections were deparaffinized and hydrated followed by heat-induced antigen retrieval with 10 mM Na-citrate buffer and blocking with 3% milk in 0.1% Tween 20. slides were stained with primary antibodies against GFP (NB600-308 Rabbit anti-GFP polyclonal from Novus Biologicals at 1:250) overnight at 4°C and then fluorescent secondary antibodies against rabbit IgG (Alexa Fluor 488 goat anti-rabbit IgG at 1:500 from a 2 mg/mL stock) for 1 hr at room temperature. RPE autofluorescence was minimized using the Vector TrueVIEW Autofluorescence Quenching Kit. Sections were counterstained with DAPI, mounted using VECTASHIELD Vibrance Antifade Mounting Medium, and imaged the same day.

#### IF - mouse eye tissue

C57BL/6 mice eyes were collected, formalin-fixed, paraffin-embedded, and sectioned into 7 μm-thick sections. Sections were deparaffinized and hydrated followed by heat-induced antigen retrieval with citrate buffer (Vector Laboratories, Inc.), permeabilized with 1% horse serum in 0.2% Triton-X, blocked with 5% Horse serum in 0.2% Triton-X, and incubated with PAX3 primary antibody (1:200 anti-rabbit, Invitrogen, 38-1801) overnight at 4°C. Samples were incubated with DyLightTM 488-labeled secondary antibody (1:2000, horse anti-rabbit. Vector Laboratories, Inc. DI-1088-1.5) for 1 hr at room temperature, treated with autofluorescence quenching kit (Vector Laboratories, Inc. SP-8400-15), and cover slips fitted using Vectashield Antifade Mounting Medium with DAPI (Vector Laboratories, Inc. H-1000-10).

#### Kaplan-Meier analysis

Zebrafish were followed for up to 70 weeks and tumor-free survival was analyzed with the Kaplan-Meier method. Tumors were defined as the first presence of a GFP-positive nodular mass at the injection site. Differences between injection groups were analyzed using log-rank statistics.

#### Single cell suspension and single-cell RNA Seq analysis

Zebrafish were euthanized with 0.4% Tricaine for 10 mins followed by immersion in ice water until no opercular movement was observed for at least 30 min as detailed in our IACUC protocol. Tumors were harvested and minced into small pieces. Minced tumors were digested for 1 hour at 37°C with collagenase and hyaluronidase in HBSS with 2% FBS (HF).

Suspensions were washed with 1:4 HF:NH_4_Cl and then further digested for 5 min with prewarmed trypsin. Following trypsin inactivation, cell suspensions were washed twice with 10% FBS in HBSS.

Cellular suspensions were loaded on a 10x Genomics Chromium instrument to generate single-cell gel beads in emulsion (GEMs). Approximately 10,000-20,000 cells were loaded per channel depending on the 10x kit used. See figures S5 and S12 for kit information per sample. Next GEM kit: targeted cell number = 10,000. Single-cell RNA-Seq libraries were prepared using Single Cell 3′ Reagent Kits v2: Chromium Single Cell 3′ Library & Gel Bead Kit v2, PN-120237; Single Cell 3′ Chip Kit v2, PN-120236; and i7 Multiplex Kit, PN-120262 (10x Genomics) and following the Single Cell 3′ Reagent Kits v2 User Guide (Manual Part # CG00052 Rev A). GEM-X kit: targeted cell number = 20,000. Single-cell RNA-Seq libraries were prepared using Chromium GEM-X Single Cell 3′ Reagent Kits v4: Library Construction Kit C, PN-1000694; Single Cell 3’ GEM Kit v4, PN-1000693; Single Cell 3’ Gel Bead Kit v4, PN-2001128; and Dual Index Kit TT Set A, PN-3000431; and following the Chromium GEM-X Single Cell 3′ Reagent Kits v4 User Guide (Manual Part # CG000731 Rev B). Libraries were sequenced on an Illumina HiSeq 4000 as 2 × 150 paired-end reads. Sequencing results were demultiplexed and converted to FASTQ format using Illumina bcl2fastq software.

A custom reference genome for the Zebrafish was constructed with GRCz11 primary assembly (Ensembl) using Cell Ranger (Cell Ranger mkref function). 10X Genomics scRNA-seq reads were then processed and aligned to this reference with Cell Ranger (Cell Ranger Count function). Further analysis and visualization was performed using Seurat (v4.1.0)^101^, cells with fewer than 200 RNA feature counts and greater than 5% mitochondrial contamination were removed with filtering (greater than 15% for wildtype eye samples). RNA counts were normalized, and FindVariableFeatures was run with the following parameters: selection.method = “vst”, nfeatures = 2,000. The cells were originally clustered in a UMAP by RunPCA then RunUMAP with dims 1:30. FindNeighbors was run with dims 1:30 followed by FindClusters using a resolution of 0.4. These methods identified up to 20 clusters in individual samples.

For comparative analysis, RNA-seq datasets were integrated together using IntegrateData with dims 1:30. New clusters were generated as before. Clusters were annotated using FindAllMarkers and the top 25 enriched genes in each cluster were compared to the Daniocell dataset^52^ and Spectacle^49^. Cluster names were given according to the closest matched cellular population between these two datasets. To determine enriched pathways, *danio rerio* gene names were converted into human Ensembl gene codes with g:Profiler^102^ and then data were analyzed with the use of QIAGEN IPA (QIAGEN Inc)^103^ and GSEA^104,105^. Pseudotime was performed using Monocle3 (v0.2.3.0)^106^.

#### Verification of sgRNAs

Zebrafish were euthanized with 0.4% Tricaine for 10 mins followed by immersion in ice water until no opercular movement was observed for at least 10 min as detailed in the IACUC protocol. Tumors were harvested and minced into small pieces. DNA was harvested according to the DNeasy Blood and Tissue Kit from Qiagen. Regions surrounding cut sites were PCR-amplified and the purity of the PCR amplicon was confirmed with agarose gel electrophoresis. Samples were cleaned up with the QIAquick PCR Purification Kit from Qiagen and sent for Sanger sequencing at the Iowa Institute of Human Genetics. Gene editing was confirmed with DECODR INDEL analysis by comparing to wildtype sequence^107^. PCR primer sequences follow:

*mitfa* PCR F: CCGGTATGTATTCACATTGTCTTG

*mitfa* PCR R: TCTTGCTTAGGATGCCTATGTATT

*ptena* PCR F: CATCCCACCAAGTGAGGTTAAAC

*ptena* PCR R: CACATACACAGTCAAGGGTGAG

*ptenb* PCR F: CAGTTCTGTTGCACCCAATAAG

*ptenb* PCR R: CTGGTGGTGTTGAGGCTATAAAG

*tp53* PCR F: AAGTATTCAGCCCCCAGGTG

*tp53* PCR R: CGCTTTTGACTCACAGTGCAAG

## Supplementary Material

1 **Figure S1:** Mitfa-deficient zebrafish have decreased tumor latency compared to wild-types by TEAZ-Skin. (**A**) Kaplan-Meier curves estimating tumor-free survival, defined as the time from electroporation until the first visible appearance of GFP+ nodular tumors, in adult wild-type zebrafish via TEAZ-Skin. Injections contained *mitfa*:Cas9 with *U6*:gRNA-*ptena* + *U6*:gRNA-*ptenb* (*ptena/b* CRISPR), *mitfa*:Cas9 with *U6*:gRNA-*tp53* (*tp53* CRISPR), *mitfa*:*GNAQ*^Q209L^-*mitfa*:GFP (GNAQ: GFP), strategy A plasmids, or strategy B plasmids (n=3 per condition). (**B**) Kaplan-Meier curves estimating tumor-free survival in adult *nacre* (*mitfa*^w2/w2^) zebrafish via TEAZ-Skin. Same injection mixes as in (A). (n=5 for Strategy B, n=3 for rest) (**B’**) Representative brightfield and fluorescent images shown at 2, 4, and 6 weeks for strategy B injection in *nacre*. (**C**) Kaplan-Meier curves estimating tumor-free survival in adult *casper* (*mitfa*^w2/w2^, *mpv17*^a9/a9^) zebrafish via TEAZ-Skin. Same injection mixes as in (A). (n=5 for Strategies A and B, n=3 for rest) (**D**) Kaplan-Meier curves estimating tumor-free survival in adult wild-type, *nacre*, and *casper* zebrafish via TEAZ-Skin. Oncogene alone (GNAQ: GFP) is compared to strategy B plasmids for each genotype. (n=5 for strategy B in *nacre* and *casper*, n=3 for rest). (**E**) Representative images of Strategy B TEAZ-Skin in wild-type and *casper* and a xanthoma that formed in a *Tg(mitfa:BRAF*^*V600E*^*); mitfa*^*w2/w2*^*; tp53*^*−/−*^ zebrafish. Note the bright orange color of the xanthoma, indicated by the black arrow.
**Figure S2:** Targeted Sanger sequencing to confirm CRISPR gene editing following TEAZ-Skin injection using Strategy B plasmids. 11 tumors and 2 wild-type tissue controls were used for INDEL analysis to verify gene editing in *tp53, ptena,* and *ptenb.* Green box indicates successful gene editing; red box indicates no gene editing. Total number of edited genes per sample are indicated in the right-most column.
**Figure S3:** Immunofluorescence of the choroid using an anti-GFP antibody in (**A**) *Tg*(*mitfa*:GFP) with 10x magnification and in (**B**) *Tg*(*mitfa*:GFP) following quenching of autofluorescence. (**C**) TEAZ-Eye–injected zebrafish expressing an eGFP construct driven by the *mitfa* promoter (*mitfa:GFP*) with 40x magnification. Sections were counterstained with DAPI to visualize nuclei.
**Figure S4:** UMAP of normal eyes and TEAZ-Eye uveal melanoma in wild-type zebrafish (Strategy B). (**A**) Integrated object is split by sequencing replicates (top) and feature plots showing *mlana* RNA expression to identify normal choroidal melanocytes and uveal melanoma cells (bottom). Color intensity (grey to red) reflects normalized average expression levels (low to high). The red dotted line indicates the melanocyte and uveal melanoma clusters.
**Figure S5:** Gross images of tissue and tumors used for single cell RNA-Seq in wild-type zebrafish.
**Figure S6:** Ingenuity Pathway Analysis of genes enriched in TEAZ-Eye compared to the tumor microenvironment. (**A**) The top 49 pathways enriched in TEAZ-Eye. (**B**) Select pathways enriched in TEAZ-Eye pertaining to fatty acid oxidation and signaling. Color intensity indicates the sign and magnitude of the z-score from blue (negative) to orange (positive). Significance is indicated by the −log(p-value).
**Figure S7:** Overall survival of patients with uveal melanoma (UM) and cutaneous melanoma (CM) based on expression levels of *FABP3*, *KIT, FABP5, LGALS2,* and *GCH*. Disease-free survival in UM and CM patients based on *FABP3, KIT, and FABP5* expression. Dotted lines represent 95% confidence intervals. Data derived from TCGA datasets.
**Figure S8:** Normalized RNA expression of *FABP5, FABP3, LGALS1, GCH, LGALS2, KIT* across the 4 TCGA molecular subclasses. ^55^ Groups were compared by ANOVA with post-hoc Tukey test. ns = not significant, * p-value<0.05; ** p-value<0.01; ***p-value<0.001; ****p-value<0.0001.
**Figure S9:** Mock injected wild-type zebrafish eyes do not show an increase in activated fibroblast markers as compared to uninjected controls. (**A**) UMAP representation of wild-type normal eyes from three different sources: mock injected, sibling-matched (3 pooled eyes), uninjected tumor-paired (2 pooled eyes), and uninjected sibling-matched (4 pooled eyes). The red dotted line indicates the fibroblast clusters. (**B**) Feature plots showing *fn1a* RNA expression across wild-type eye control groups. Color intensity (grey to red) reflects normalized average expression levels (low to high). (**C**) Dot plot of fibroblast activation marker genes in the fibroblast clusters of wild-type eye control groups. Dot size indicates the percentage of cells expressing each gene; color intensity (grey to blue) reflects normalized average expression levels (low to high).
**Figure S10:** Ingenuity Pathway Analysis of enriched genes in TEAZ-Eye and TEAZ-Skin compared to the combined tumor microenvironment (eye and skin). (**A**) Enriched pathways from genes that were common to only TEAZ-Eye and TEAZ-Skin compared to the combined tumor microenvironment. Families of pathways have been identified with a colored box and colored label including: cancer proliferation (green), WNT signaling (blue), metabolism (red), PTEN regulation (black), and RAF/MAPK signaling (black). (**B**) Enriched pathways from genes that were common to all four gene subsets (TEAZ-Eye, TEAZ-Skin, choroidal melanocytes, skin melanocytes) versus their respective combined microenvironments (normal or tumor). Families of pathways have been identified with a colored box and colored label including: melanocyte identity (black), signals downstream of GNAQ/GNA11 (blue), and survival/proliferation (green). Color intensity indicates the sign and magnitude of the z-score from blue (negative) to orange (positive). Significance is indicated by the −log(p-value).
**Figure S11:** Melanocyte rescue experiments in *nacre* zebrafish by TEAZ-Eye using plasmids containing *mitfa*:*mitfa*; *mitfa*:GFP (miniCoopR^29^). (**A-A’**) H&E images (10x and 40x magnification) of wild-type (*AB*) eyes following TEAZ-Eye injection with (**A**) control *mitfa*-GFP or (**A’**) miniCoopR plasmids. (**B-B’**) H&E images (10x and 40x magnification) of *mitfa*^*w2/w2*^ (*nacre*) eyes following TEAZ-Eye injection with (**B**) control *mitfa*-GFP or (**B’**) miniCoopR plasmids, rescued melanocytes in the choroid are indicated by a red asterisk. (**C**) Representative dorsal image of a transgenic *Tg(mitfa:GFP); mitfa*^*w2/w2*^ injected with *mitfa*:*GNAQ*^*Q209L*^ following TEAZ-Eye injection.
**Figure S12:** Gross images of tissue and tumors used for single cell RNA-Seq in *casper* zebrafish and transgenic *Tg*(*mitfa*:GFP); *mitfa*^+/w2^ and *Tg*(*mitfa*:GFP); *mitfa*^w2/w2^ whole embryos.
**Figure S13:** UMAP representation of UM tumors derived from *casper* zebrafish, with accompanying pseudotime analysis. (**A**) UMAP representation following scRNA-seq on normal *casper* control eyes (3 pooled eyes) and UM tumors (3 pooled tumors) derived in *casper* (TEAZ-Eye) (**B**) Feature plot showing *eGFP* expression and *mitfa*^w2/w2^ expression (**C**) UMAP following re-clustering of *mitfa*^w2/w2^ positive cells from (B), with overlayed unsupervised pseudotime analysis using Monocle analysis.
**Figure S14:** Immunofluorescent staining for Pax3 in the mouse limbus. (**A**) Representative 20x image of the mouse eye showing Pax3 positive staining in the limbus region. Sections were counterstained with DAPI to visualize nuclei. (**B**) Post-image processing showing the retina (red), cornea (orange) and limbus (purple), Pax3-positive cells in green.
**Figure S15:** Representative images of TEAZ-Skin induced tumors (*Strategy B*) in (**A**) wild-type at 16 weeks, (**B**) conditional *mitfa*-KO (wild-type) at 8 weeks and (**C**) *casper* zebrafish at 8 weeks post TEAZ-Skin. Brightfield and GFP-overlaid images are shown to highlight primary and metastatic cells in (B-C), and the absence of metastasis in (A).
**Figure S16:** Violin plots representing *mitfa* expression as well as pigmentation genes in BRAF^V600E^- and GNAQ^Q209L^-positive tumors by single cell RNA-Seq analysis.
**Figure S17:** Dot plot of genes enriched in chromatoblasts and in uveal melanoma tumors derived in wild-type zebrafish following TEAZ-Eye compared to the tumor microenvironment (TME). Genes enriched in a particular pigment cell type are labelled, including: iridophore, xanthophore, and melanocyte.
**Figure S18:** UMAP representation of melanocytes and melanocyte progenitors in *casper* normal eyes and in the three sequencing replicates of wild-type normal eyes. The red dotted line indicates melanocyte progenitors in the *casper* sample and melanocytes that are clustering closely with such progenitors in wild-type samples.

**Supplemental Table 1** – Findallmarkers of the integrated object between wild-type control eyes and wild-type TEAZ-Eye.

**Supplemental Table 2** – RNA expression data of *FABP3*, *FABP5, LGALS2, LGALS1, BSCL2, GCH1,* and *KIT* across the 4 TCGA molecular subclasses in uveal melanoma with corresponding sample IDs.

**Supplemental Table 3** – Differentially expressed genes between cancer associated fibroblasts from wild-type TEAZ-Eye and normal fibroblasts from wild-type normal eyes.

**Supplemental Table 4** – Findmarkers for choroidal melanocytes, skin melanocytes, TEAZ-Eye, and TEAZ-Skin versus their respective microenvironments. Table also includes gene names in every intersection of the Venn Diagram comparison of all four groups.

**Supplemental Table 5** – Differentially expressed genes between eye and skin melanocytes and between TEAZ-Eye and TEAZ-Skin, respectively.

**Supplemental Table 6** – Findallmarkers of the *casper* TEAZ-Eye single-cell object.

**Supplemental Table 7** – Findallmarkers of the *casper* normal eye single-cell object.

**Supplemental Table 8** – Findallmarkers of the integrated object between *casper* TEAZ-Eye and *casper* normal eye with cluster names labelled.

**Supplemental Table 9** – Findallmarkers of the subset of GFP-positive clusters from the integrated object between *casper* TEAZ-Eye and *casper* normal eye.

**Supplemental Table 10** – Findallmarkers of the integrated object of neural crest and neural crest-derived cells from *Tg(mitfa:GFP) mitfa*^*+lw2*^ and Tg(mitfa:GFP) *mitfa*^w2/w2^ embryos.

**Supplemental Table 11** – Differentially expressed genes between neural crest and neural crest-derived clusters from *Tg(mitfa:GFP) mitfa*^*+lw2*^ and Tg(mitfa:GFP) *mitfa*^*w2/w2*^ embryos.

**Supplemental Table 12** – Injection and tumor penetrance of wild-type and *casper* zebrafish injected with Strategy B plasmids and either *U6*:gRNA-nontargeting or *U6*:gRNA-*mitfa*.

**Supplemental Table 13** – Differentially expressed genes between BRAF^V600E^-driven tumors and *GNAQ*^Q209L^-driven tumors in adult zebrafish.

## Highlights

- Choroid-targeted electroporation of oncogenic GNAQ induces anatomically correct UM in adult zebrafish.
- Mitfa-independent melanocyte progenitor cells are highly susceptible to GNAQ-driven transformation.
- Germline loss of *mitfa* accelerates UM onset via progenitor enrichment, a phenotype not recapitulated by conditional *mitfa*-KO in adult zebrafish.
- Oncogenic BRAF and GNAQ transform molecularly and developmentally distinct subpopulations of cells within the melanocyte lineage.
