# Supplementary material for: Mitfa-Independent Melanocyte Progenitors are Highly Susceptible to GNAQ-induced Uveal Melanoma in Adult Zebrafish
Source: bioRxiv. 2025 Sep 9:2025.05.05.652300. Preprint. [Version 3] doi: 10.1101/2025.05.05.652300 (PMC12431003; doi:10.1101/2025.05.05.652300)
Supplement: 1 — Figure S1: Mitfa-deficient zebrafish have decreased tumor latency compared to wild-types by TEAZ-Skin. (A) Kaplan-Meier curves estimating tumor-free survival, defined as the time from electroporation until the first visible appearance of GFP+ nodular tumors, in adult wild-type zebrafish via TEAZ-Skin. Injections contained mitfa:Cas9 with U6:gRNA-ptena + U6:gRNA-ptenb (ptena/b CRISPR), mitfa:Cas9 with U6:gRNA-tp53 (tp53 CRISPR), mitfa:GNAQQ209L-mitfa:GFP (GNAQ: GFP), strategy A plasmids, or strategy B plasmids (n=3 per condition). (B) Kaplan-Meier curves estimating tumor-free survival in adult nacre (mitfaw2/w2) zebrafish via TEAZ-Skin. Same injection mixes as in (A). (n=5 for Strategy B, n=3 for rest) (B’) Representative brightfield and fluorescent images shown at 2, 4, and 6 weeks for strategy B injection in nacre. (C) Kaplan-Meier curves estimating tumor-free survival in adult casper (mitfaw2/w2, mpv17a9/a9) zebrafish via TEAZ-Skin. Same injection mixes as in (A). (n=5 for Strategies A and B, n=3 for rest) (D) Kaplan-Meier curves estimating tumor-free survival in adult wild-type, nacre, and casper zebrafish via TEAZ-Skin. Oncogene alone (GNAQ: GFP) is compared to strategy B plasmids for each genotype. (n=5 for strategy B in nacre and casper, n=3 for rest). (E) Representative images of Strategy B TEAZ-Skin in wild-type and casper and a xanthoma that formed in a Tg(mitfa:BRAFV600E); mitfaw2/w2; tp53−/− zebrafish. Note the bright orange color of the xanthoma, indicated by the black arrow. Figure S2: Targeted Sanger sequencing to confirm CRISPR gene editing following TEAZ-Skin injection using Strategy B plasmids. 11 tumors and 2 wild-type tissue controls were used for INDEL analysis to verify gene editing in tp53, ptena, and ptenb. Green box indicates successful gene editing; red box indicates no gene editing. Total number of edited genes per sample are indicated in the right-most column. Figure S3: Immunofluorescence of the choroid using an anti-GFP antibody in (A) T [file NIHPP2025.05.05.652300v3-supplement-1.pdf]

bioRxiv preprint doi: https://doi.org/10.1101/2025.05.05.652300; this version posted September 9, 2025. The copyright holder for this preprint (which was not certified by peer review) is the author/funder, who has granted bioRxiv a license to display the preprint in perpetuity. It is made available under aCC-BY 4.0 International license.

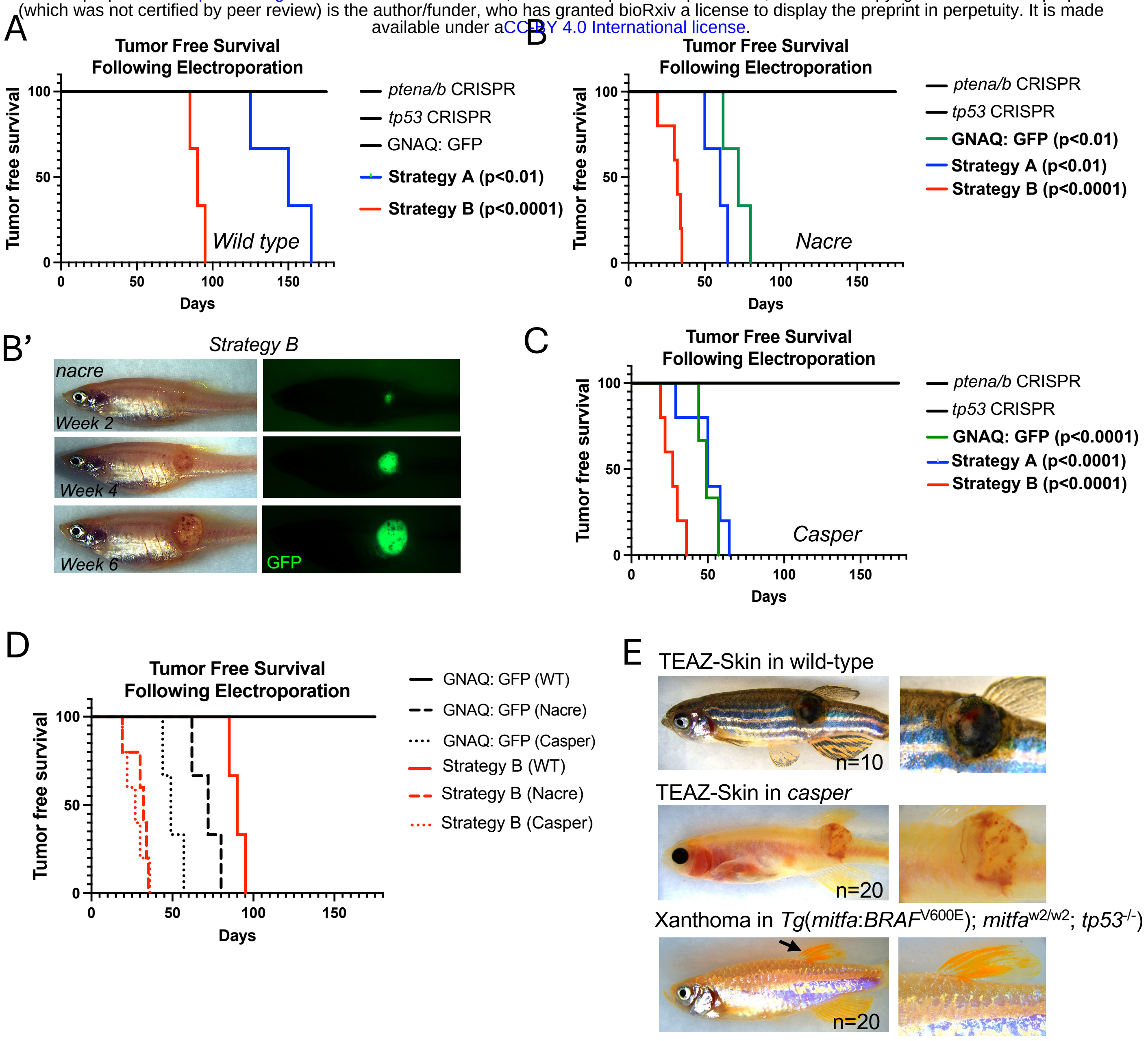

FIGURE S1

bioRxiv preprint doi: https://doi.org/10.1101/2025.05.05.652300; this version posted September 9, 2025. The copyright holder for this preprint (which was not certified by peer review) is the author/funder, who has granted bioRxiv a license to display the preprint in perpetuity. It is made available under aCC-BY 4.0 International license.

Tumor #	ptena	ptenb	tp53	# of edited genes
1	CRISPR editing	CRISPR editing	CRISPR editing	3
2	CRISPR editing	CRISPR editing	CRISPR editing	3
3	No editing	CRISPR editing	CRISPR editing	2
4	CRISPR editing	CRISPR editing	CRISPR editing	3
5	CRISPR editing	CRISPR editing	CRISPR editing	3
6	CRISPR editing	CRISPR editing	CRISPR editing	3
7	CRISPR editing	CRISPR editing	CRISPR editing	3
8	CRISPR editing	CRISPR editing	CRISPR editing	3
9	CRISPR editing	CRISPR editing	CRISPR editing	3
10	CRISPR editing	CRISPR editing	CRISPR editing	3
11	CRISPR editing	CRISPR editing	CRISPR editing	3
**Germline**	**ptena**	**ptenb**	**tp53**	
WT1	No editing	No editing	No editing	0
WT2	No editing	No editing	No editing	0

Green: CRISPR editing
Red: No editing

FIGURE S2

bioRxiv preprint doi: https://doi.org/10.1101/2025.05.05.652300; this version posted September 9, 2025. The copyright holder for this preprint (which was not certified by peer review) is the author/funder, who has granted bioRxiv a license to display the preprint in perpetuity. It is made available under aCC-BY 4.0 International license.

A

Tg(mitfa-GFP) Zebrafish Line

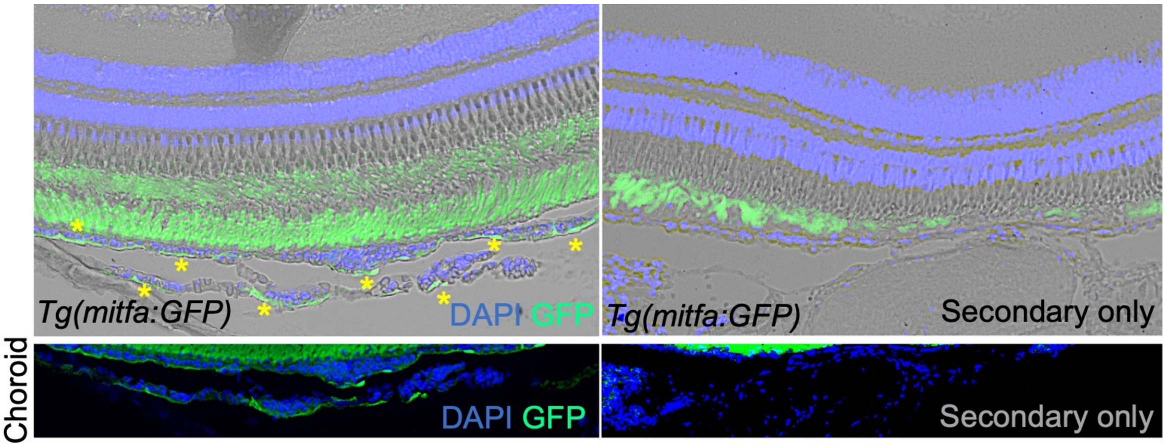

B

Tg(mitfa-GFP) Zebrafish Line

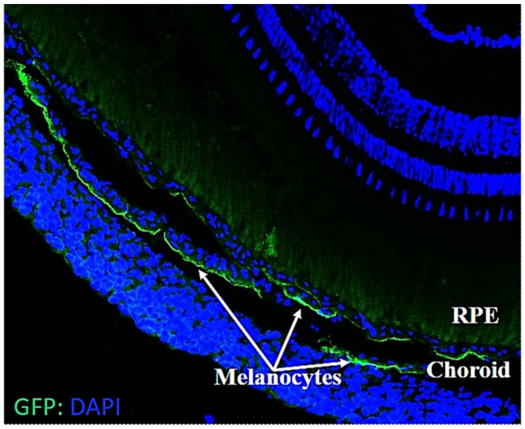

Anti-GFP IF

C

TEAZ-Eye using *mitfa:GFP* plasmids into WT Zebrafish

Choroid

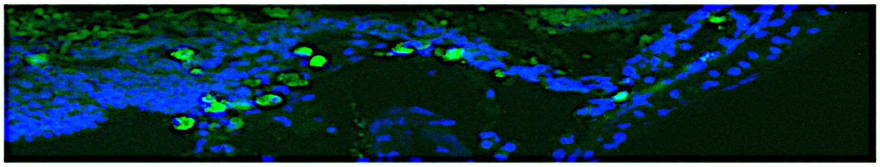

Anti-GFP IF

Choroid

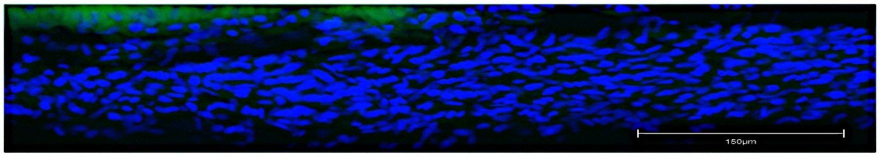

Secondary-only Control

FIGURE S3

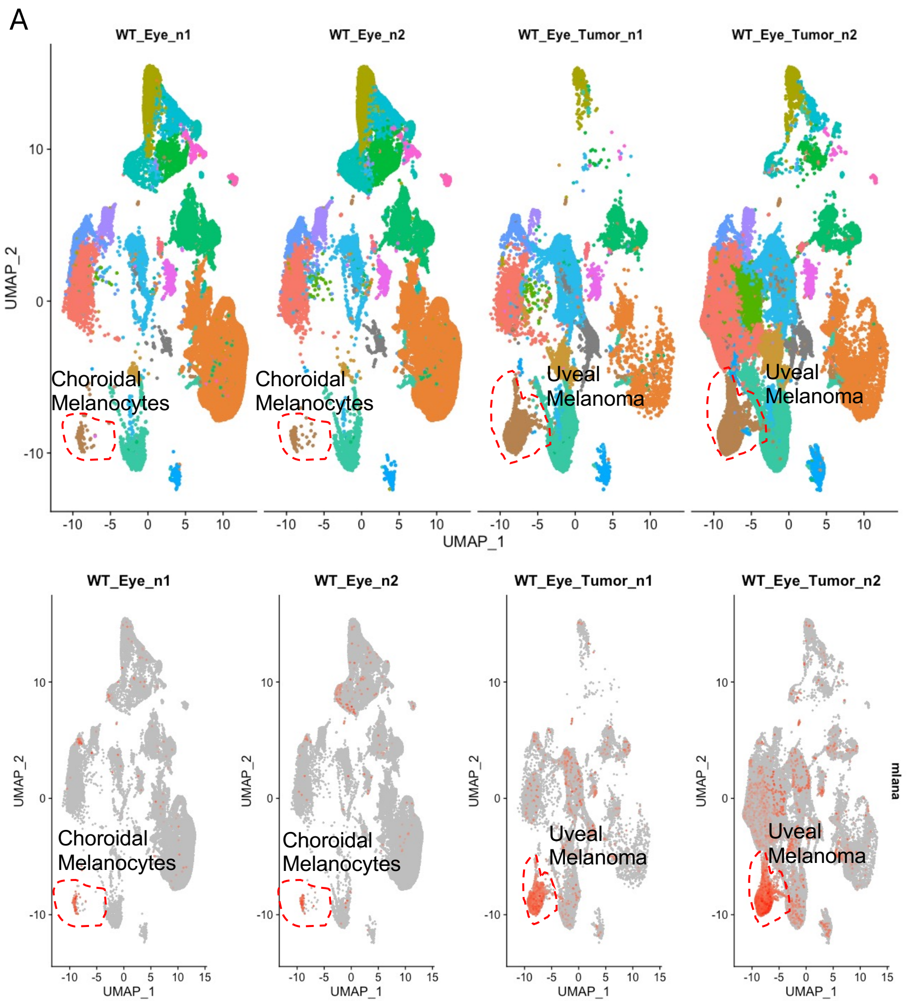
A
WT_Eye_n1
WT_Eye_n2
WT_Eye_Tumor_n1
WT_Eye_Tumor_n2
UMAP_2
UMAP_1
Choroidal Melanocytes
Choroidal Melanocytes
Uveal Melanoma
Uveal Melanoma
WT_Eye_n1
WT_Eye_n2
WT_Eye_Tumor_n1
WT_Eye_Tumor_n2
Choroidal Melanocytes
Choroidal Melanocytes
Uveal Melanoma
Uveal Melanoma
mlana

FIGURE S4

Single-cell RNA seq Replicates

Tissue	Genotype	Replicate 1 (pooled tissue, tumors)	Replicate 2 (pooled tissue, tumors)
Skin	Wild-type	1 skin sample 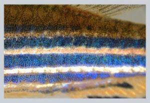	1 skin sample 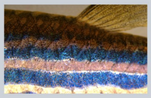
Skin Tumor	Wild-type	1 tumor sample 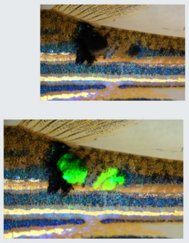	1 tumor sample 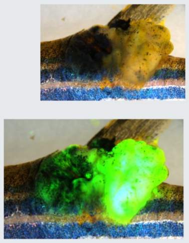
Eye Paired (tumor matched bilateral eye)	Wild-type	2 pooled eyes 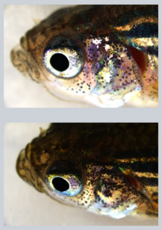	
Eye sibling matched	Wild-type	4 pooled eyes 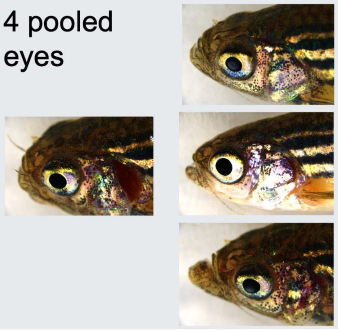	
Eye mock injected, sibling matched	Wild-type	3 pooled eyes 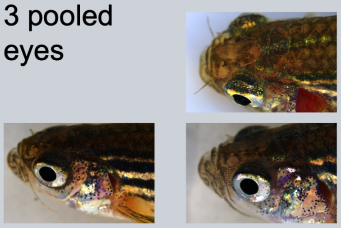	
Eye Tumor	Wild-type	1 tumor sample 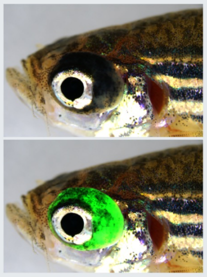	2 pooled tumor samples 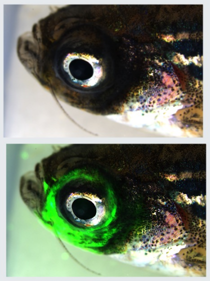 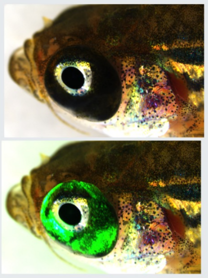

FIGURE S5

A

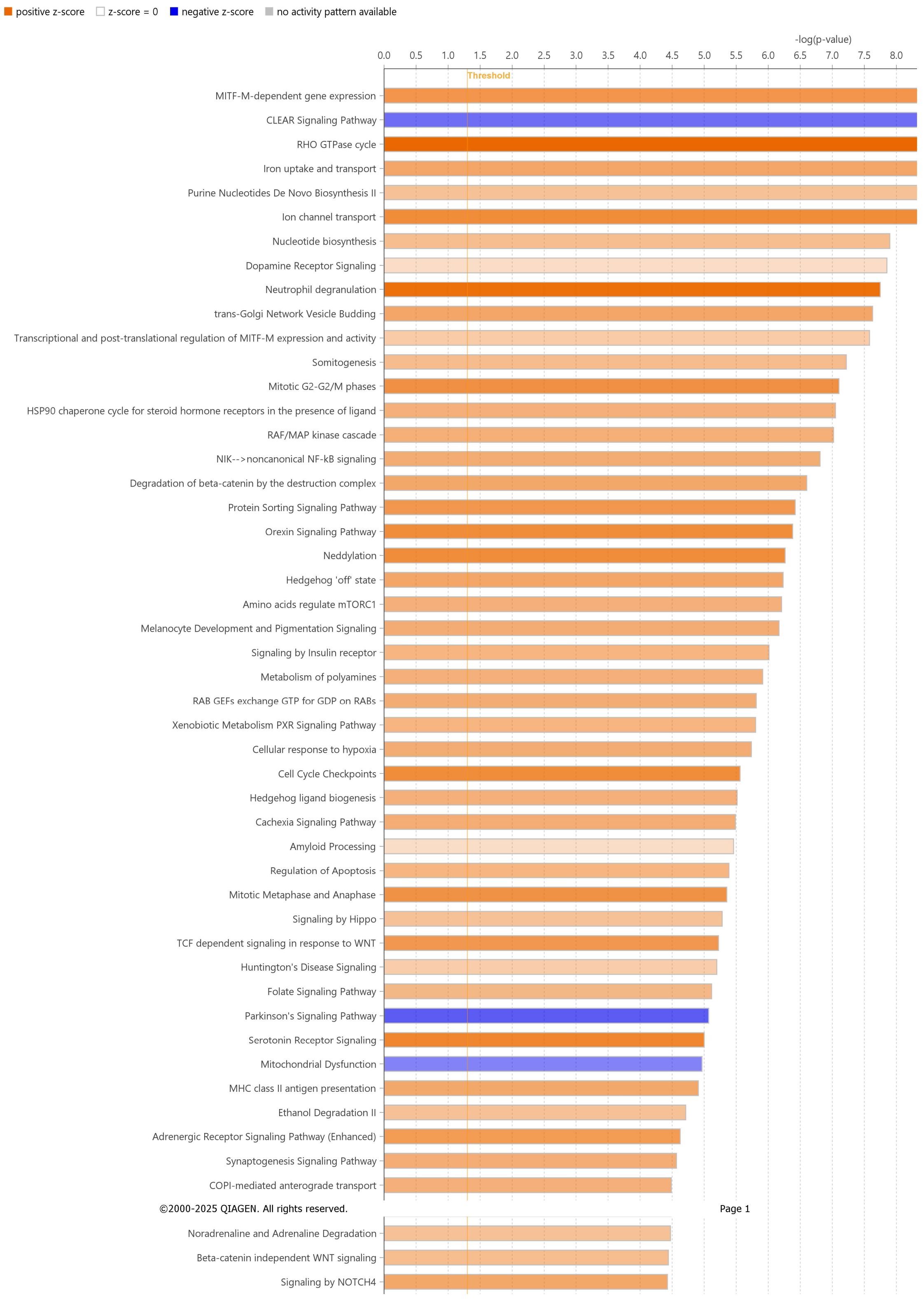
positive z-score
z-score = 0
negative z-score
no activity pattern available
-log(p-value)
0.0 0.5 1.0 1.5 2.0 2.5 3.0 3.5 4.0 4.5 5.0 5.5 6.0 6.5 7.0 7.5 8.0
Threshold
MITF-M-dependent gene expression
CLEAR Signaling Pathway
RHO GTPase cycle
Iron uptake and transport
Purine Nucleotides De Novo Biosynthesis II
Ion channel transport
Nucleotide biosynthesis
Dopamine Receptor Signaling
Neutrophil degranulation
trans-Golgi Network Vesicle Budding
Transcriptional and post-translational regulation of MITF-M expression and activity
Somitogenesis
Mitotic G2-G2/M phases
HSP90 chaperone cycle for steroid hormone receptors in the presence of ligand
RAF/MAP kinase cascade
NIK-->noncanonical NF-kB signaling
Degradation of beta-catenin by the destruction complex
Protein Sorting Signaling Pathway
Orexin Signaling Pathway
Neddylation
Hedgehog 'off' state
Amino acids regulate mTORC1
Melanocyte Development and Pigmentation Signaling
Signaling by Insulin receptor
Metabolism of polyamines
RAB GEFs exchange GTP for GDP on RABs
Xenobiotic Metabolism PXR Signaling Pathway
Cellular response to hypoxia
Cell Cycle Checkpoints
Hedgehog ligand biogenesis
Cachexia Signaling Pathway
Amyloid Processing
Regulation of Apoptosis
Mitotic Metaphase and Anaphase
Signaling by Hippo
TCF dependent signaling in response to WNT
Huntington's Disease Signaling
Folate Signaling Pathway
Parkinson's Signaling Pathway
Serotonin Receptor Signaling
Mitochondrial Dysfunction
MHC class II antigen presentation
Ethanol Degradation II
Adrenergic Receptor Signaling Pathway (Enhanced)
Synaptogenesis Signaling Pathway
COPI-mediated anterograde transport
©2000-2025 QIAGEN. All rights reserved.
Page 1
Noradrenaline and Adrenaline Degradation
Beta-catenin independent WNT signaling
Signaling by NOTCH4

B

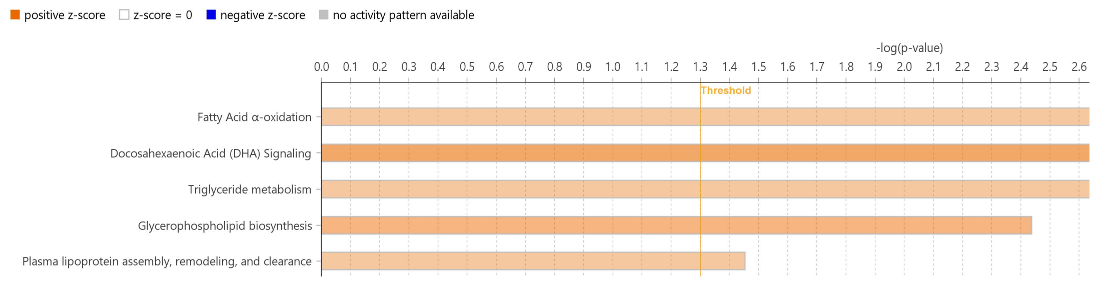
positive z-score
z-score = 0
negative z-score
no activity pattern available
-log(p-value)
0.0 0.1 0.2 0.3 0.4 0.5 0.6 0.7 0.8 0.9 1.0 1.1 1.2 1.3 1.4 1.5 1.6 1.7 1.8 1.9 2.0 2.1 2.2 2.3 2.4 2.5 2.6
Threshold
Fatty Acid α-oxidation
Docosahexaenoic Acid (DHA) Signaling
Triglyceride metabolism
Glycerophospholipid biosynthesis
Plasma lipoprotein assembly, remodeling, and clearance

FIGURE S6

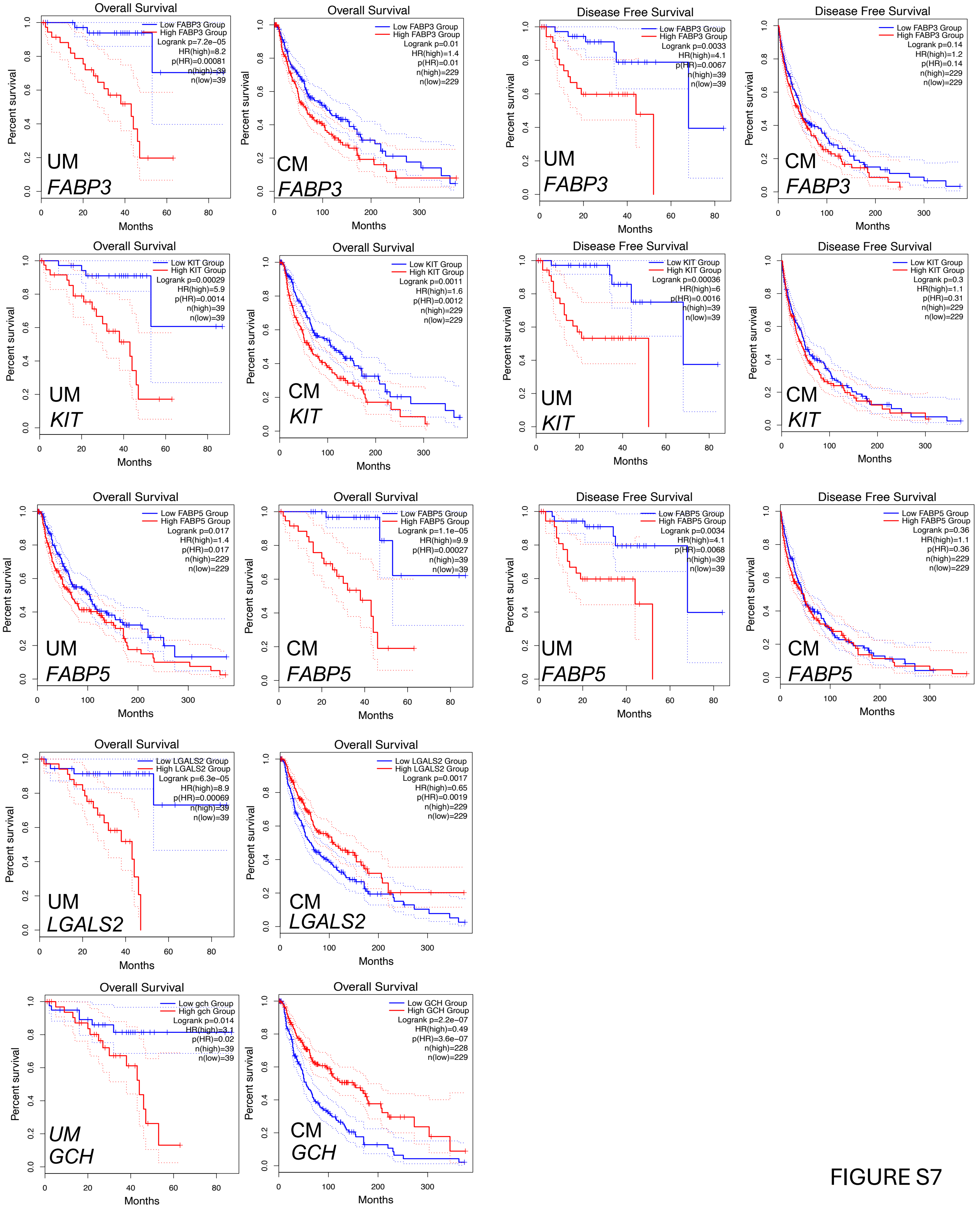

Overall Survival
Low FABP3 Group
High FABP3 Group
Logrank p=7.2e−05
HR(high)=8.2
p(HR)=0.00081
n(high)=39
n(low)=39
UM
FABP3
Overall Survival
Low FABP3 Group
High FABP3 Group
Logrank p=0.01
HR(high)=1.4
p(HR)=0.01
n(high)=229
n(low)=229
CM
FABP3
Disease Free Survival
Low FABP3 Group
High FABP3 Group
Logrank p=0.0033
HR(high)=4.1
p(HR)=0.0067
n(high)=39
n(low)=39
UM
FABP3
Disease Free Survival
Low FABP3 Group
High FABP3 Group
Logrank p=0.14
HR(high)=1.2
p(HR)=0.14
n(high)=229
n(low)=229
CM
FABP3
Overall Survival
Low KIT Group
High KIT Group
Logrank p=0.00029
HR(high)=5.9
p(HR)=0.0014
n(high)=39
n(low)=39
UM
KIT
Overall Survival
Low KIT Group
High KIT Group
Logrank p=0.0011
HR(high)=1.6
p(HR)=0.0012
n(high)=229
n(low)=229
CM
KIT
Disease Free Survival
Low KIT Group
High KIT Group
Logrank p=0.00036
HR(high)=6
p(HR)=0.0016
n(high)=39
n(low)=39
UM
KIT
Disease Free Survival
Low KIT Group
High KIT Group
Logrank p=0.3
HR(high)=1.1
p(HR)=0.31
n(high)=229
n(low)=229
CM
KIT
Overall Survival
Low FABP5 Group
High FABP5 Group
Logrank p=0.017
HR(high)=1.4
p(HR)=0.017
n(high)=229
n(low)=229
UM
FABP5
Overall Survival
Low FABP5 Group
High FABP5 Group
Logrank p=1.1e−05
HR(high)=9.9
p(HR)=0.00027
n(high)=39
n(low)=39
CM
FABP5
Disease Free Survival
Low FABP5 Group
High FABP5 Group
Logrank p=0.0034
HR(high)=4.1
p(HR)=0.0068
n(high)=39
n(low)=39
UM
FABP5
Disease Free Survival
Low FABP5 Group
High FABP5 Group
Logrank p=0.36
HR(high)=1.1
p(HR)=0.36
n(high)=229
n(low)=229
CM
FABP5
Overall Survival
Low LGALS2 Group
High LGALS2 Group
Logrank p=6.3e−05
HR(high)=8.9
p(HR)=0.00069
n(high)=39
n(low)=39
UM
LGALS2
Overall Survival
Low LGALS2 Group
High LGALS2 Group
Logrank p=0.0017
HR(high)=0.65
p(HR)=0.0019
n(high)=229
n(low)=229
CM
LGALS2
Overall Survival
Low gch Group
High gch Group
Logrank p=0.014
HR(high)=3.1
p(HR)=0.02
n(high)=39
n(low)=39
UM
GCH
Overall Survival
Low GCH Group
High GCH Group
Logrank p=2.2e−07
HR(high)=0.49
p(HR)=3.6e−07
n(high)=228
n(low)=229
CM
GCH
Percent survival
Months

FIGURE S7

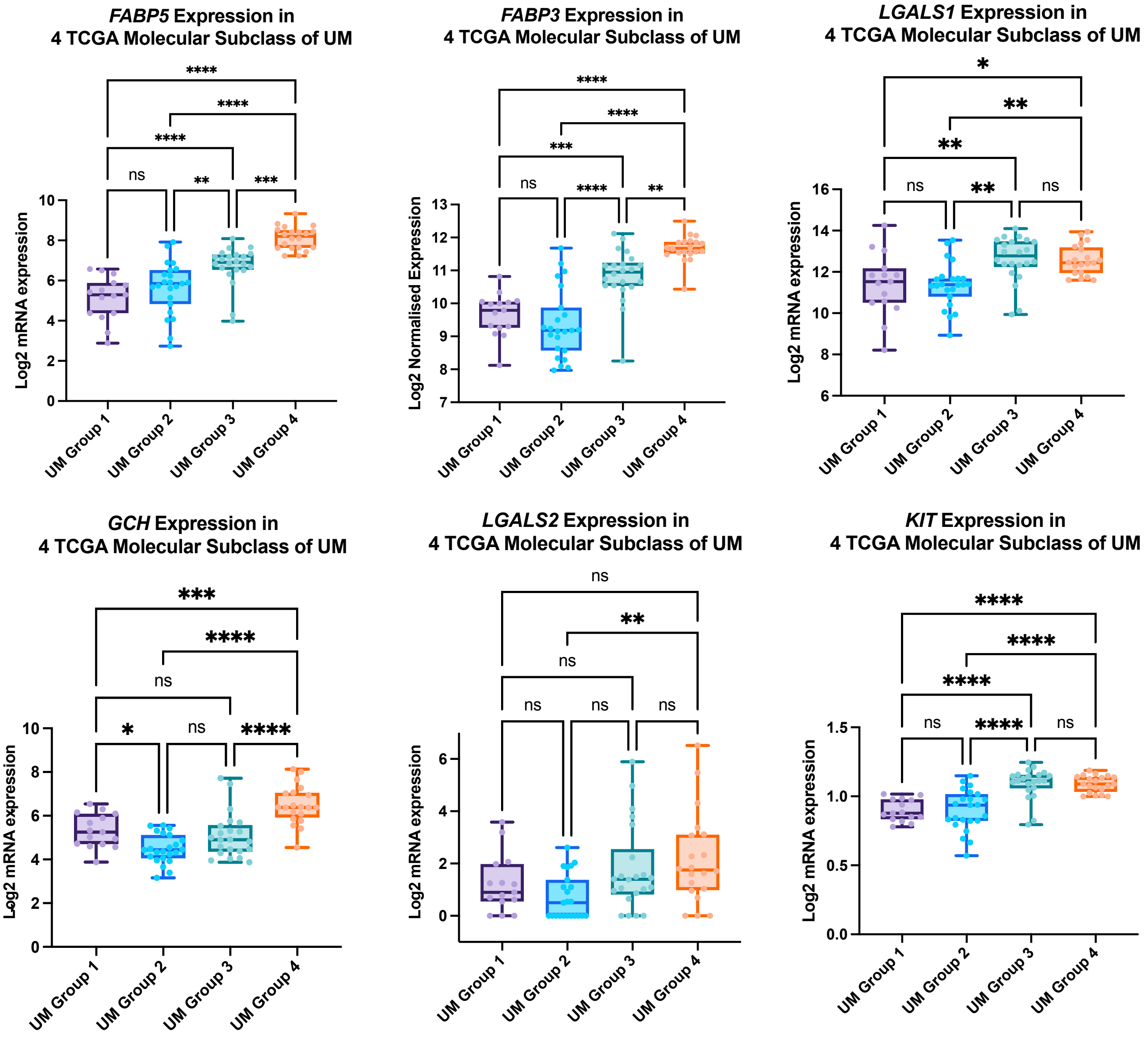
FABP5 Expression in
4 TCGA Molecular Subclass of UM
Log2 mRNA expression
FABP3 Expression in
4 TCGA Molecular Subclass of UM
Log2 Normalised Expression
LGALS1 Expression in
4 TCGA Molecular Subclass of UM
Log2 mRNA expression
GCH Expression in
4 TCGA Molecular Subclass of UM
Log2 mRNA expression
LGALS2 Expression in
4 TCGA Molecular Subclass of UM
Log2 mRNA expression
KIT Expression in
4 TCGA Molecular Subclass of UM
Log2 mRNA expression
UM Group 1
UM Group 2
UM Group 3
UM Group 4

FIGURE S8

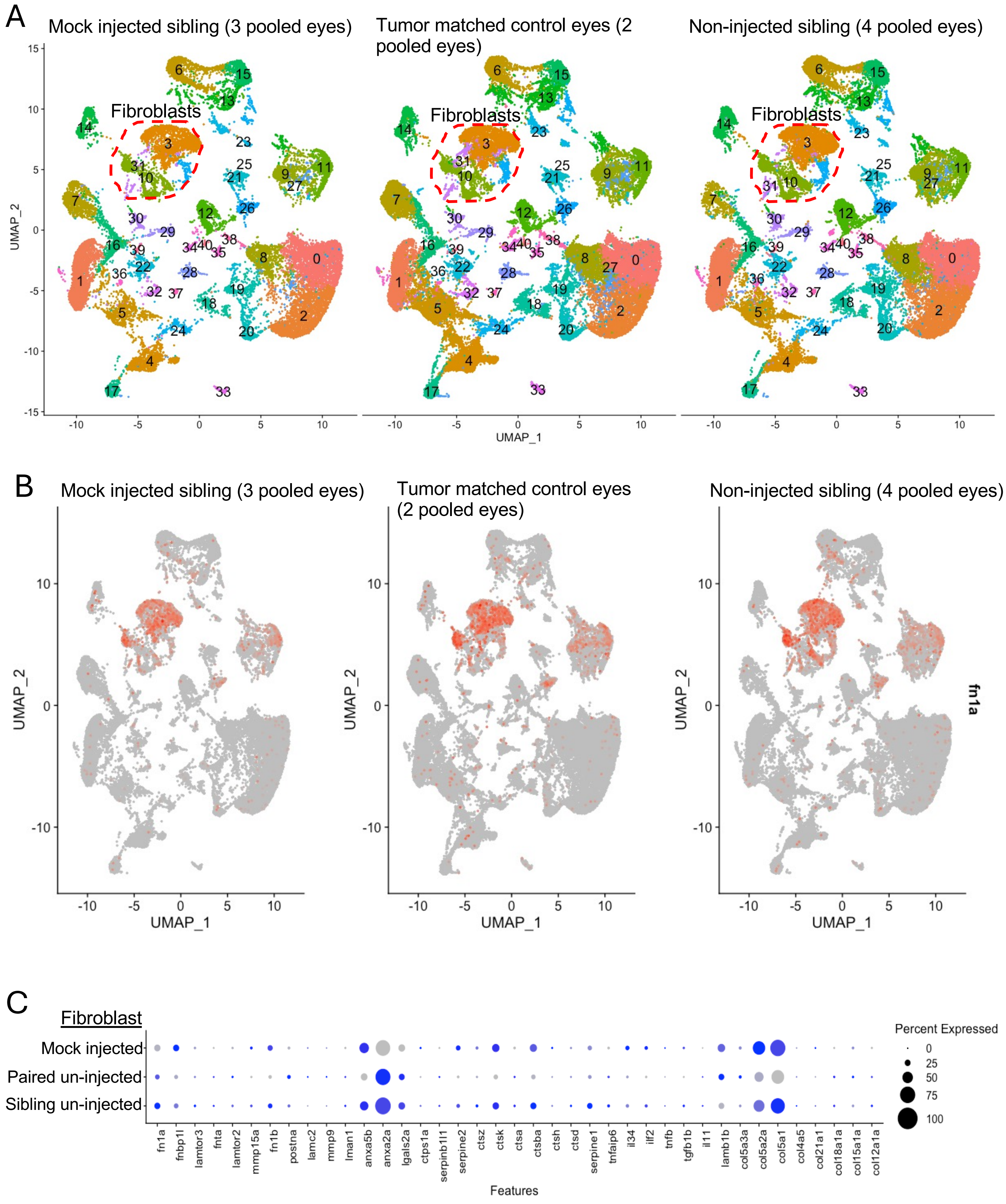
A
Mock injected sibling (3 pooled eyes)
Tumor matched control eyes (2 pooled eyes)
Non-injected sibling (4 pooled eyes)
Fibroblasts
UMAP_1
UMAP_2
B
Mock injected sibling (3 pooled eyes)
Tumor matched control eyes (2 pooled eyes)
Non-injected sibling (4 pooled eyes)
fn1a
UMAP_1
UMAP_2
C
Fibroblast
Mock injected
Paired un-injected
Sibling un-injected
fn1a
fnbp1l
lamtor3
fnta
lamtor2
mmp15a
fn1b
postna
lamc2
mmp9
lman1
anxa5b
anxa2a
lgals2a
ctps1a
serpinb1l1
serpine2
ctsz
ctsk
ctsa
ctsba
ctsh
ctsd
serpine1
tnfaip6
il34
ilf2
tnfb
tgfb1b
il11
lamb1b
col5a3a
col5a2a
col5a1
col4a5
col21a1
col18a1a
col15a1a
col12a1a
Features
Percent Expressed
0
25
50
75
100

FIGURE S9

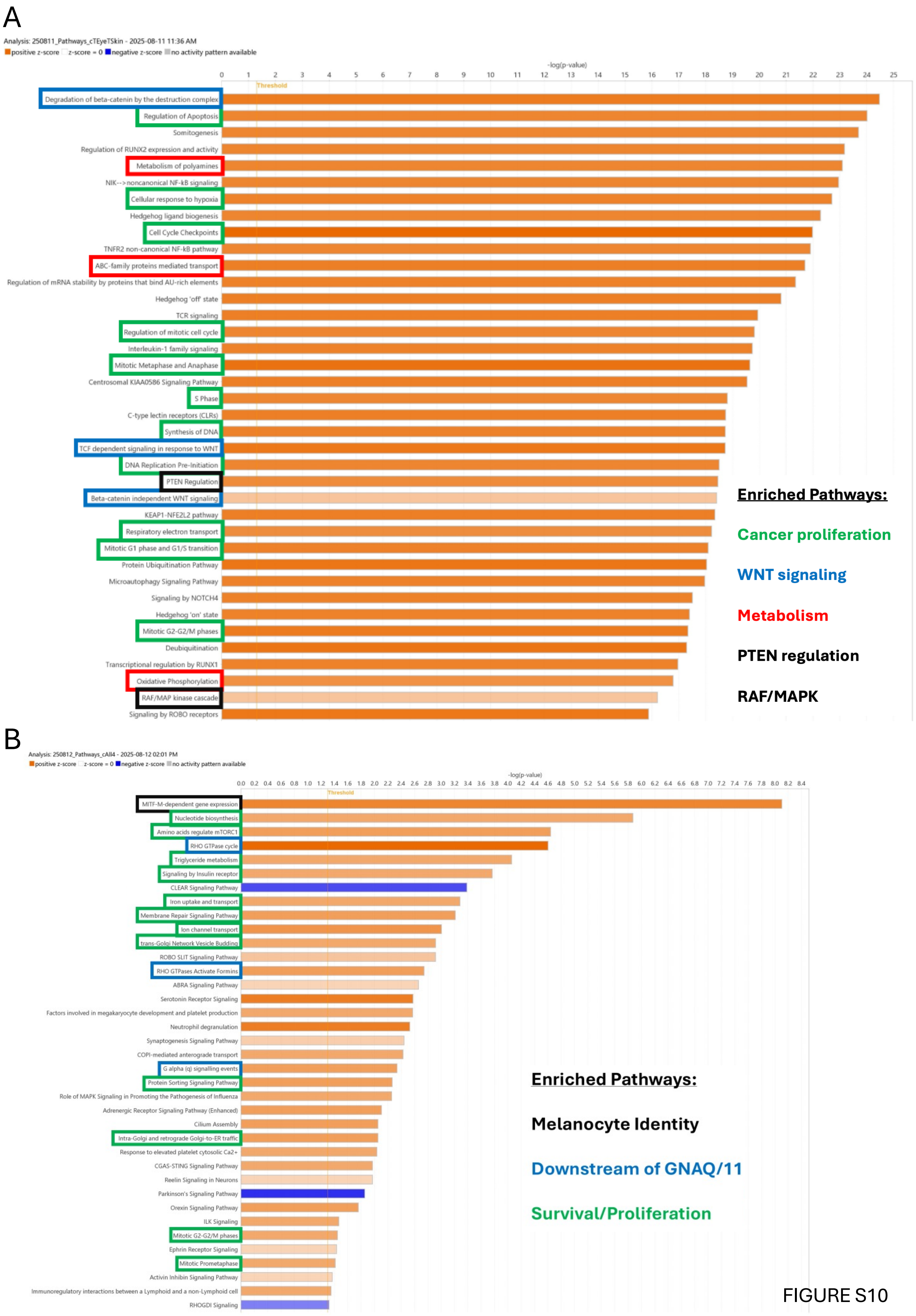

A
Analysis: 250811_Pathways_cTEyeTSkin - 2025-08-11 11:36 AM
positive z-score z-score = 0 negative z-score no activity pattern available
-log(p-value)
Threshold
Degradation of beta-catenin by the destruction complex
Regulation of Apoptosis
Somitogenesis
Regulation of RUNX2 expression and activity
Metabolism of polyamines
NIK-->noncanonical NF-kB signaling
Cellular response to hypoxia
Hedgehog ligand biogenesis
Cell Cycle Checkpoints
TNFR2 non-canonical NF-kB pathway
ABC-family proteins mediated transport
Regulation of mRNA stability by proteins that bind AU-rich elements
Hedgehog 'off' state
TCR signaling
Regulation of mitotic cell cycle
Interleukin-1 family signaling
Mitotic Metaphase and Anaphase
Centrosomal KIAA0586 Signaling Pathway
S Phase
C-type lectin receptors (CLRs)
Synthesis of DNA
TCF dependent signaling in response to WNT
DNA Replication Pre-Initiation
PTEN Regulation
Beta-catenin independent WNT signaling
KEAP1-NFE2L2 pathway
Respiratory electron transport
Mitotic G1 phase and G1/S transition
Protein Ubiquitination Pathway
Microautophagy Signaling Pathway
Signaling by NOTCH4
Hedgehog 'on' state
Mitotic G2-G2/M phases
Deubiquitination
Transcriptional regulation by RUNX1
Oxidative Phosphorylation
RAF/MAP kinase cascade
Signaling by ROBO receptors
Enriched Pathways:
Cancer proliferation
WNT signaling
Metabolism
PTEN regulation
RAF/MAPK
B
Analysis: 250812_Pathways_cAll4 - 2025-08-12 02:01 PM
positive z-score z-score = 0 negative z-score no activity pattern available
-log(p-value)
Threshold
MITF-M-dependent gene expression
Nucleotide biosynthesis
Amino acids regulate mTORC1
RHO GTPase cycle
Triglyceride metabolism
Signaling by Insulin receptor
CLEAR Signaling Pathway
Iron uptake and transport
Membrane Repair Signaling Pathway
Ion channel transport
trans-Golgi Network Vesicle Budding
ROBO SLIT Signaling Pathway
RHO GTPases Activate Formins
ABRA Signaling Pathway
Serotonin Receptor Signaling
Factors involved in megakaryocyte development and platelet production
Neutrophil degranulation
Synaptogenesis Signaling Pathway
COPI-mediated anterograde transport
G alpha (q) signalling events
Protein Sorting Signaling Pathway
Role of MAPK Signaling in Promoting the Pathogenesis of Influenza
Adrenergic Receptor Signaling Pathway (Enhanced)
Cilium Assembly
Intra-Golgi and retrograde Golgi-to-ER traffic
Response to elevated platelet cytosolic Ca2+
CGAS-STING Signaling Pathway
Reelin Signaling in Neurons
Parkinson's Signaling Pathway
Orexin Signaling Pathway
ILK Signaling
Mitotic G2-G2/M phases
Ephrin Receptor Signaling
Mitotic Prometaphase
Activin Inhibin Signaling Pathway
Immunoregulatory interactions between a Lymphoid and a non-Lymphoid cell
RHOGDI Signaling
Enriched Pathways:
Melanocyte Identity
Downstream of GNAQ/11
Survival/Proliferation

FIGURE S10

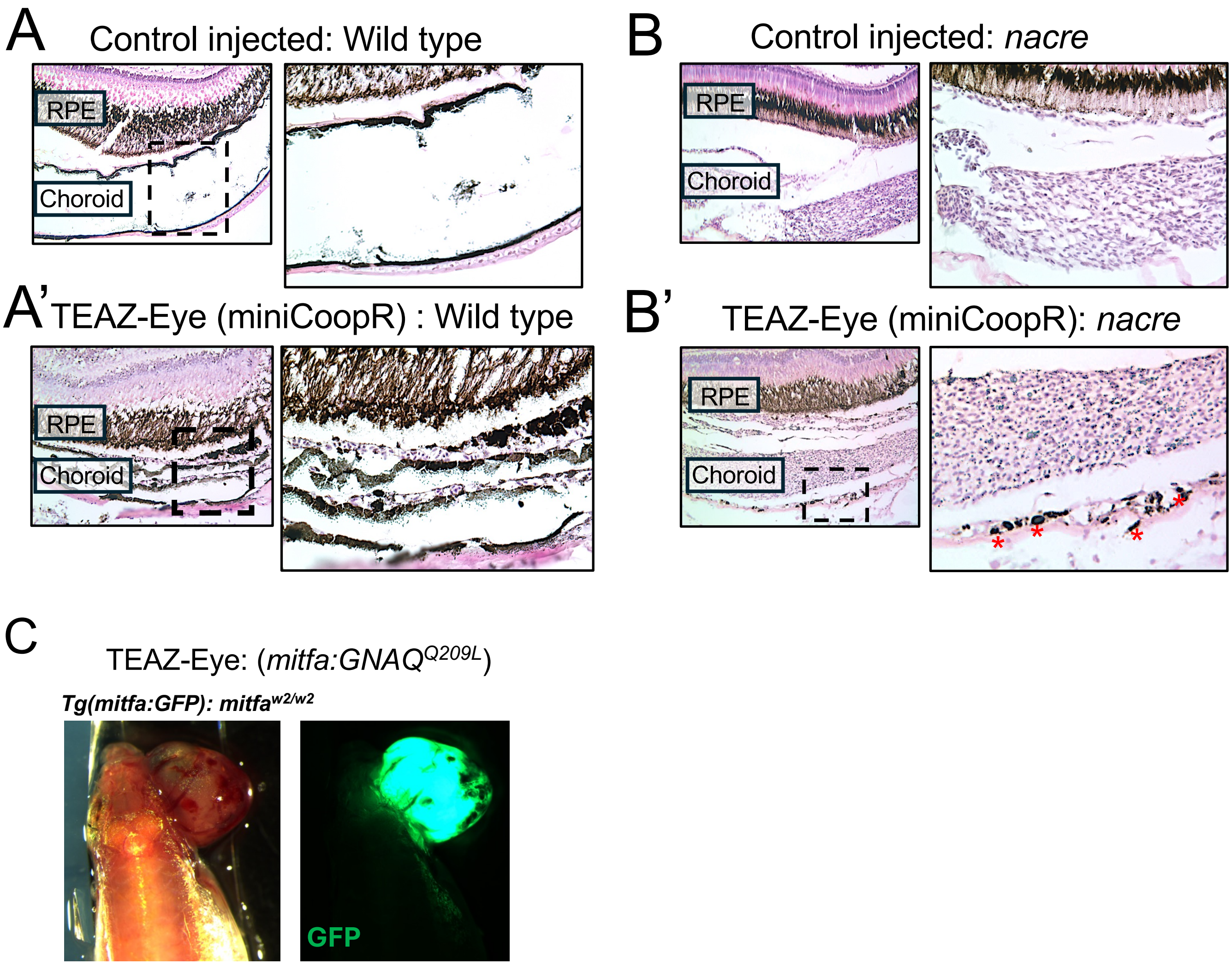
A
Control injected: Wild type
RPE
Choroid
B
Control injected: nacre
RPE
Choroid
A’
TEAZ-Eye (miniCoopR) : Wild type
RPE
Choroid
B’
TEAZ-Eye (miniCoopR): nacre
RPE
Choroid
*
*
*
*
C
TEAZ-Eye: (mitfa:GNAQQ209L)
Tg(mitfa:GFP): mitfaw2/w2
GFP

Figure S11

Single-cell RNA seq Replicates

Tissue	Genotype	Replicate 1 (pooled tissue, tumors)
Eye	Casper	3 pooled eyes 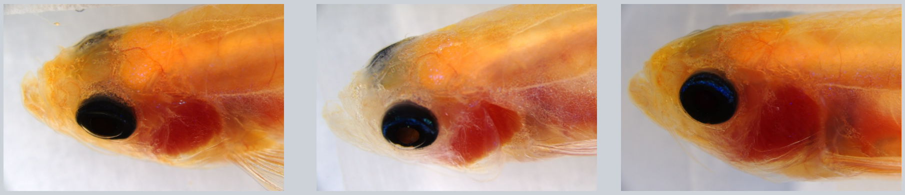
Eye Tumor	Casper	3 pooled tumors 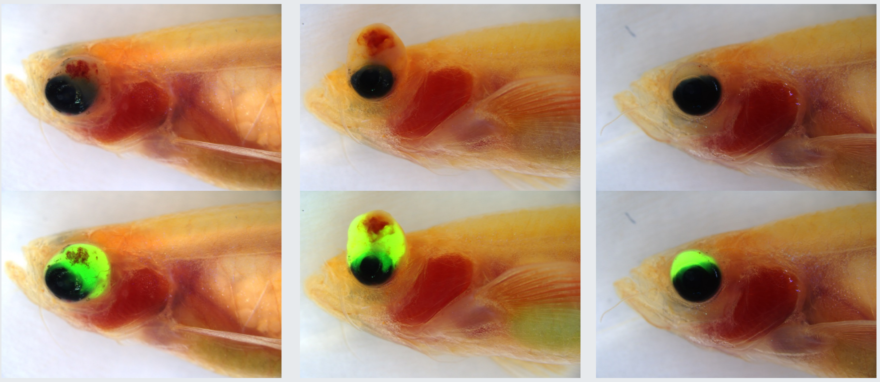
Whole embryo 28hpf	Wild-type Tg(mitfa:GFP); $mitfa^{+/w2}$	Pooled embryos 25 total 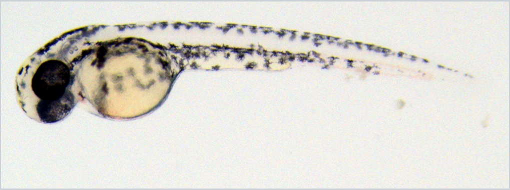
Whole embryo 28hpf	Nacre Tg(mitfa:GFP); $mitfa^{w2/w2}$	Pooled embryos 25 total 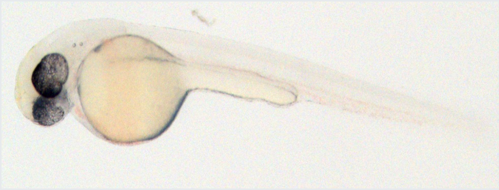

Figure S12

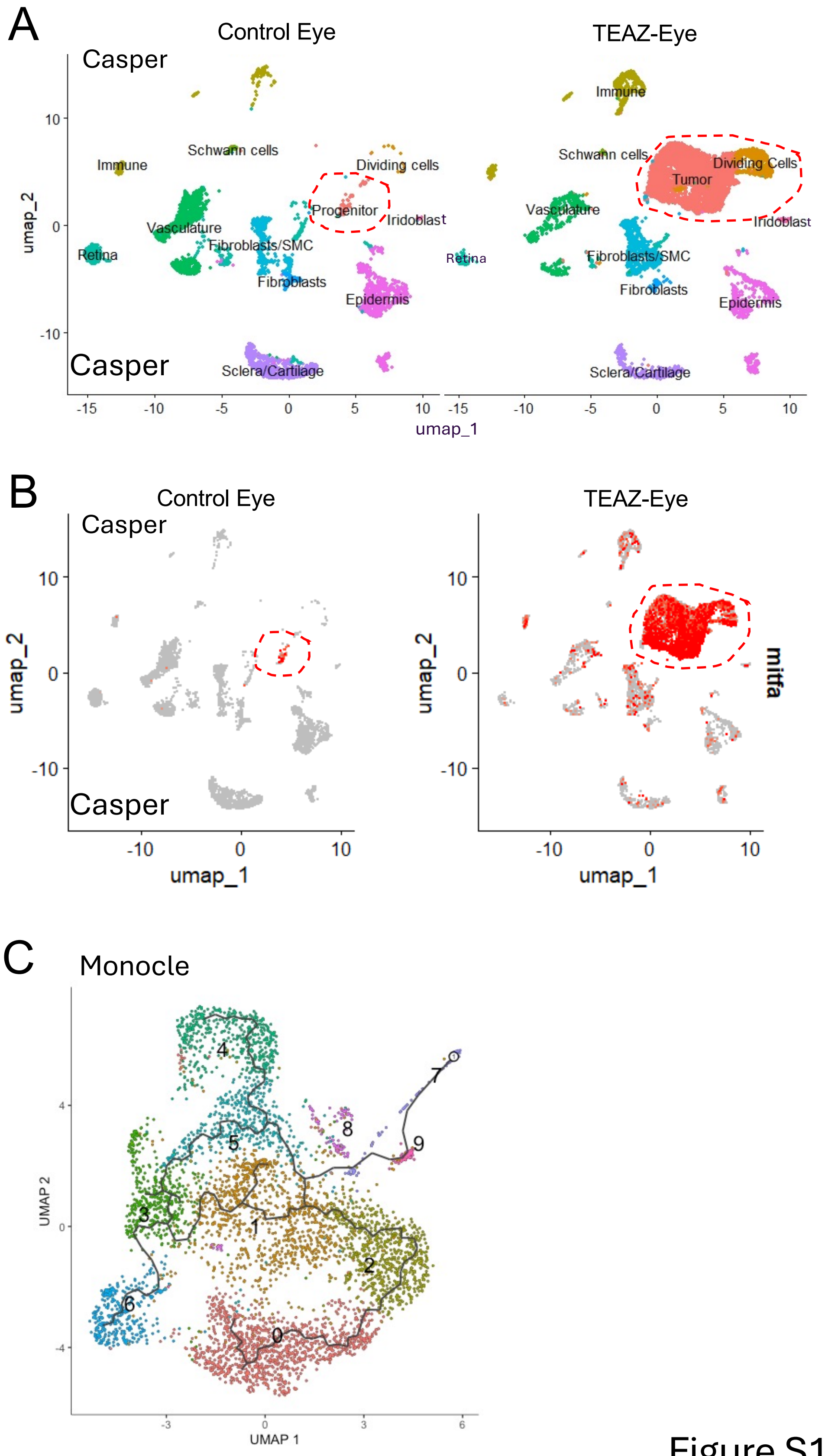
A
Control Eye
TEAZ-Eye
Casper
Immune
Schwann cells
Dividing cells
Progenitor
Iridoblast
Vasculature
Fibroblasts/SMC
Retina
Fibroblasts
Epidermis
Sclera/Cartilage
Tumor
Dividing Cells
umap_1
umap_2
B
mitfa
C
Monocle
UMAP 1
UMAP 2

Figure S13

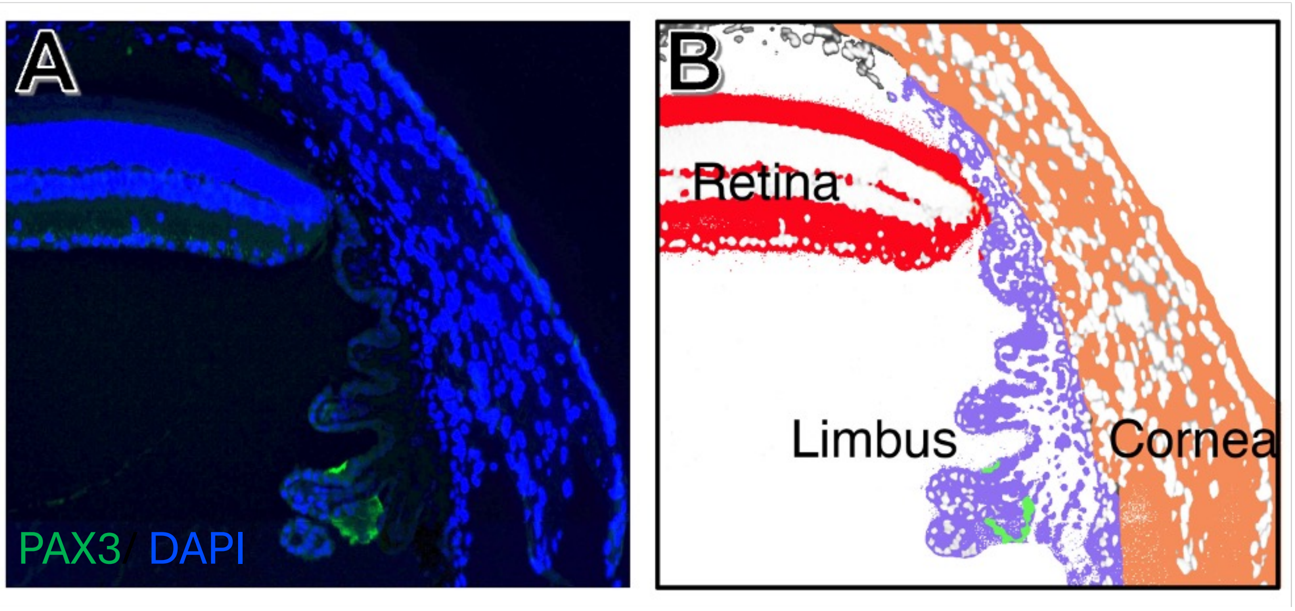

FIGURE S14

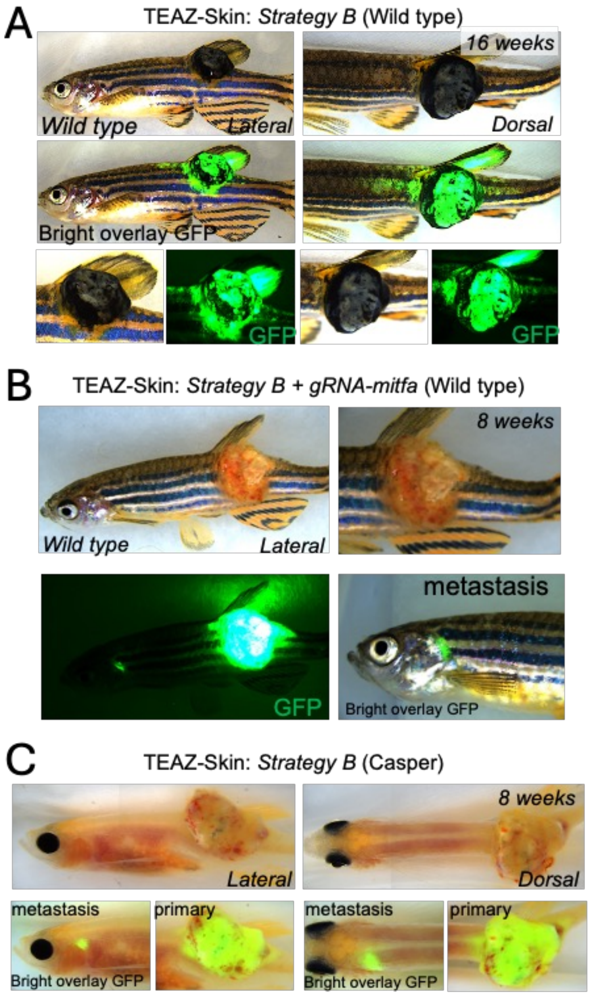
A
TEAZ-Skin: Strategy B (Wild type)
16 weeks
Wild type
Lateral
Dorsal
Bright overlay GFP
GFP
GFP
B
TEAZ-Skin: Strategy B + gRNA-mitfa (Wild type)
8 weeks
Wild type
Lateral
metastasis
GFP
Bright overlay GFP
C
TEAZ-Skin: Strategy B (Casper)
8 weeks
Lateral
Dorsal
metastasis
primary
Bright overlay GFP
metastasis
primary
Bright overlay GFP

Figure S15

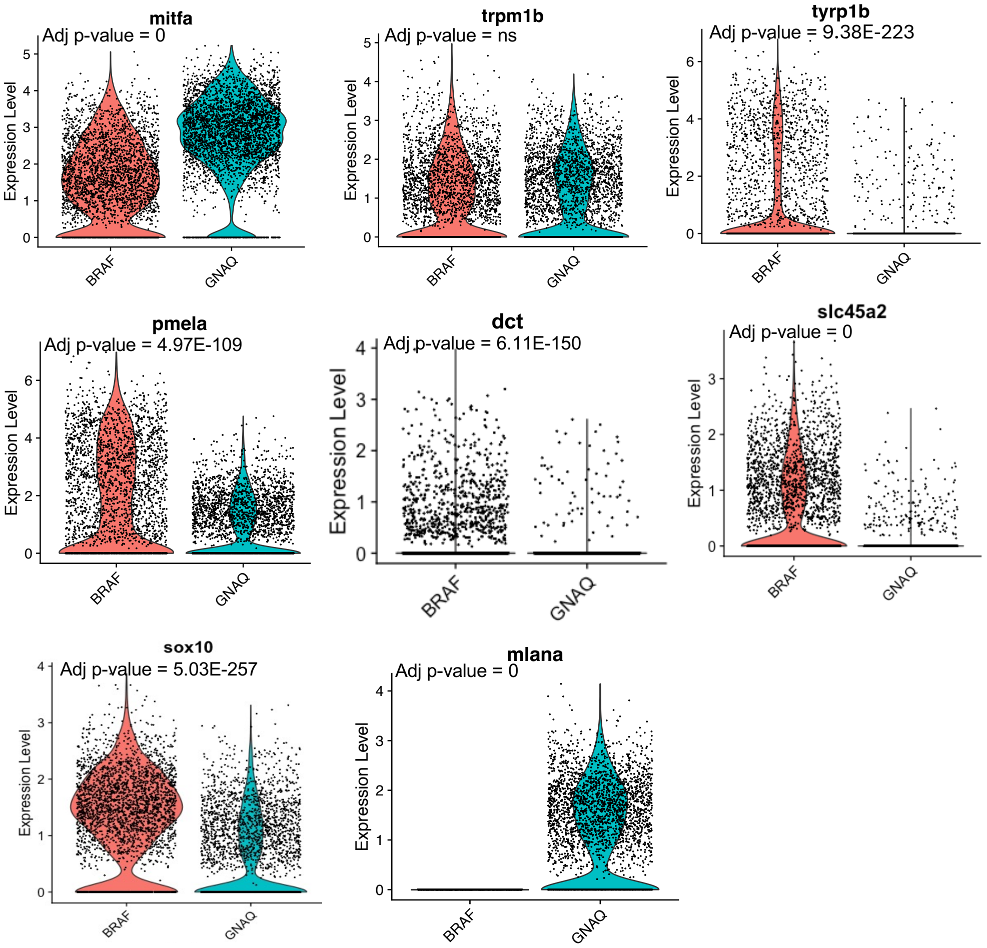
mitfa
Adj p-value = 0
trpm1b
Adj p-value = ns
tyrp1b
Adj p-value = 9.38E-223
pmela
Adj p-value = 4.97E-109
dct
Adj p-value = 6.11E-150
slc45a2
Adj p-value = 0
sox10
Adj p-value = 5.03E-257
mlana
Adj p-value = 0
Expression Level
BRAF
GNAQ

Figure S16

TEAZ-Eye Strategy B (Wild-type)

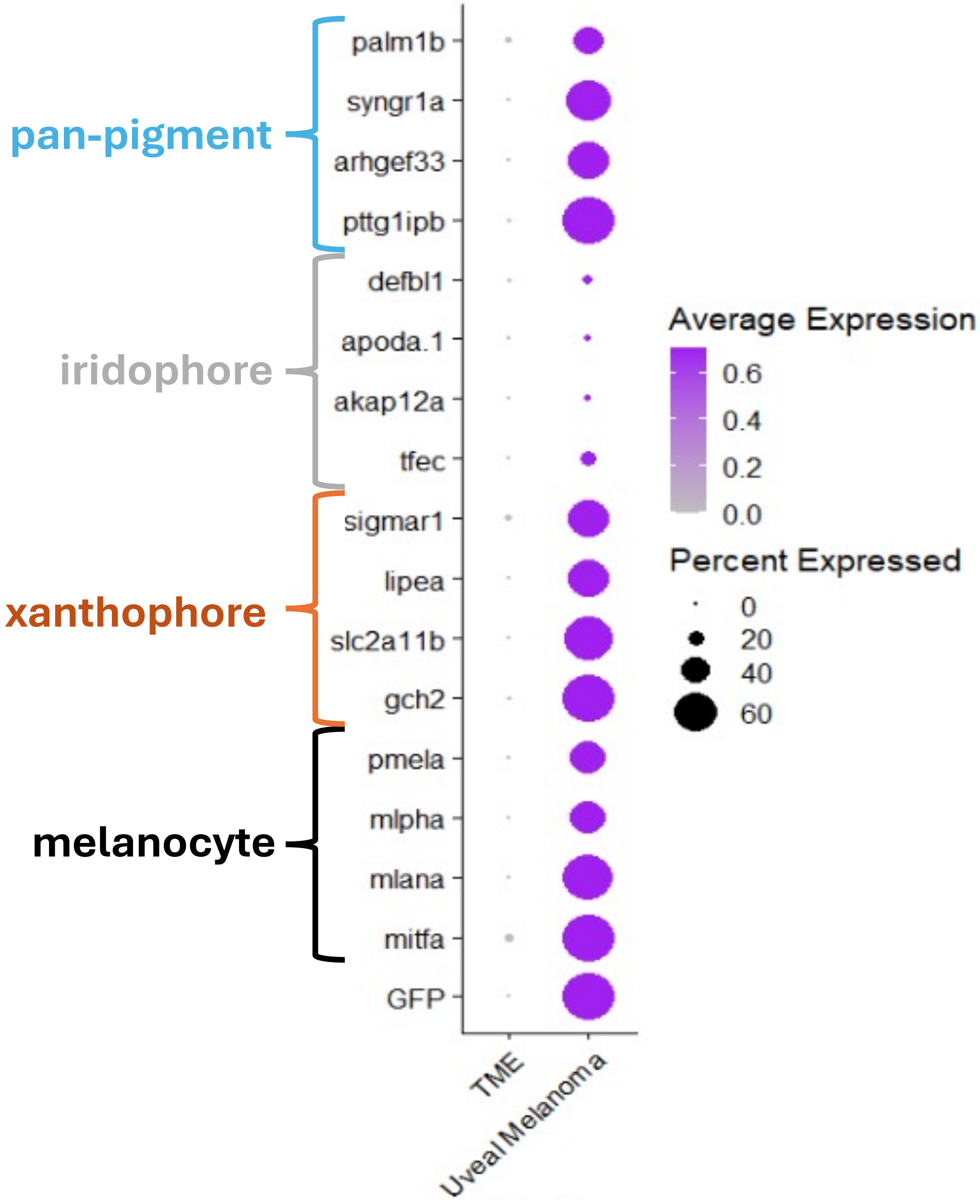

Figure S17

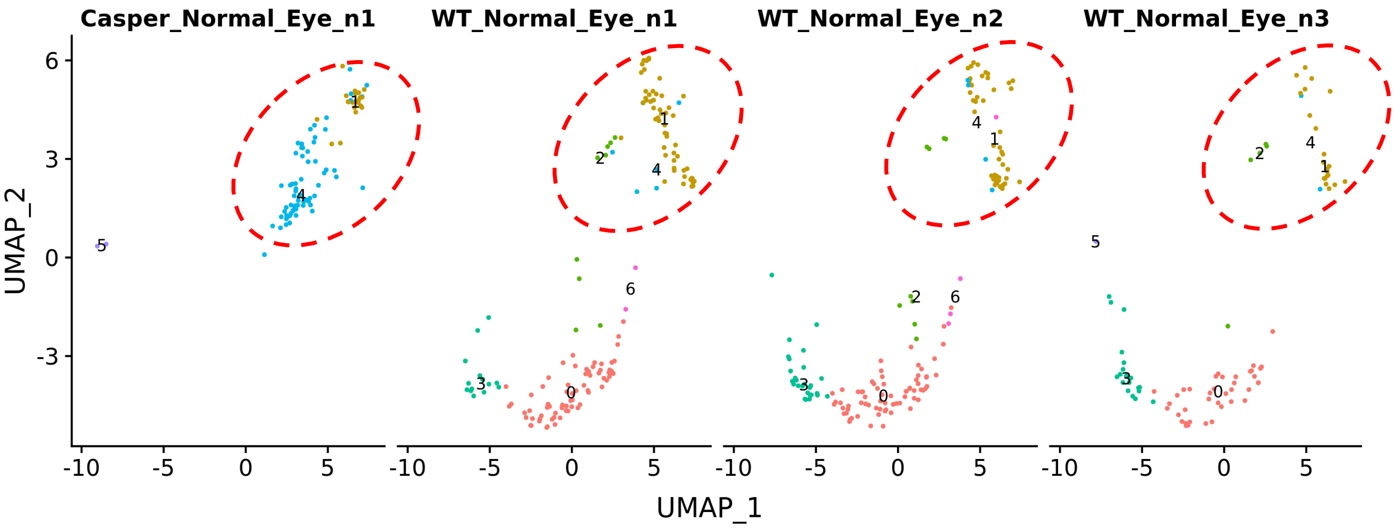
Casper_Normal_Eye_n1
WT_Normal_Eye_n1
WT_Normal_Eye_n2
WT_Normal_Eye_n3
UMAP_2
UMAP_1
6
3
0
-3
-10
-5
0
5

Figure S18
